# Predictive accuracy of changes in the inferior vena cava diameter for predicting fluid responsiveness in patients with sepsis: A systematic review and meta-analysis

**DOI:** 10.1371/journal.pone.0310462

**Published:** 2025-05-09

**Authors:** Hao Zhang, Jingyuan Jiang, Min Dai, Yan Liang, Ningxiang Li, Yongli Gao

**Affiliations:** 1 Department of Emergency Medicine, West China Hospital, Sichuan University/West China School of Nursing, Sichuan University, Chengdu, Sichuan, China; 2 Institute of Disaster Medicine, Sichuan University, Chengdu, Sichuan, China; 3 Nursing Key Laboratory of Sichuan Province, Chengdu, Sichuan, China; Scuola Superiore Sant'Anna, ITALY

## Abstract

**Background:**

Existing guidelines emphasize the importance of initial fluid resuscitation therapy in sepsis management. However, in previous meta-analyses, there have been inconsistencies in differentiating between spontaneously breathing and mechanically ventilated septic patients.

**Objective:**

To consolidate the literature on the predictive accuracy of changes in the inferior vena cava diameter (∆IVC) for fluid responsiveness in septic patients.

**Methods:**

The Embase, Web of Science, Cochrane Library, MEDLINE, PubMed, Wanfang, China National Knowledge Infrastructure (CNKI), Chinese Biomedical (CBM) and VIP (Weipu) databases were comprehensively searched. Statistical analyses were performed with Stata 15.0 software and Meta-DiSc 1.4.

**Results:**

Twenty-one research studies were deemed suitable for inclusion. The sensitivity and specificity of ∆ IVC were 0.84 (95% CI 0.76, 0.90) and 0.87 (95% CI 0.80, 0.91), respectively. With respect to the distensibility of the inferior vena cava (dIVC), the sensitivity was 0.79 (95% CI 0.68, 0.86), and the specificity was 0.82 (95% CI 0.73, 0.89). For collapsibility of the inferior vena cava (cIVC), the sensitivity and specificity values were 0.92 (95% CI 0.83, 0.96) and 0.93 (95% CI 0.86, 0.97), respectively.

**Conclusion:**

The results indicated that ∆IVC is as a dependable marker for fluid responsiveness in sepsis patients. dIVC and cIVC also exhibited high levels of accuracy in predicting fluid responsiveness in septic patients.

## Introduction

A 2017 study published in *The Lancet* reported that there were over 48 million cases of sepsis worldwide [1], with a 90-day mortality rate of 35.5% among septic ICU patients [2], resulting in an estimated 5.3 million deaths annually [3]. Sepsis and septic shock, which cause severe acute impairment and deterioration of survivors’ chronic health status, have emerged as significant global public health concerns [4,5]. Existing guidelines [6–8] emphasize the importance of initial fluid resuscitation therapy in sepsis management, as this treatment increases cardiac output, enhances organ and tissue perfusion, and reduces mortality [9]. Previous studies have shown that, either alone or in combination, static assessment indicators of fluid responsiveness are not sufficient to accurately and dynamically assess the fluid responsiveness of fluid resuscitation in septic patients, although they can be the most intuitive clinical judgment through physical examination[10,11].The existing guidelines recommend evaluating the patient’s circulatory status via dynamic indicators during the initial fluid resuscitation procedure to select additional treatment choices [6–8]. However, determining the optimal volume and target endpoints for fluid resuscitation remains challenging for frontline intensivists in clinical practice [12]. Excessive fluid replacement can lead to serious complications, including tissue edema, hypoxia, and cardiac failure, which can exacerbate clinical outcomes and potentially increase mortality [13,14]. Thus, accurate, objective, and effective dynamic index monitoring methods are essential for evaluating fluid responsiveness in septic patients.

Point-of-care ultrasound (POCUS) is essential for managing sepsis volume because of its convenient and noninvasive monitoring capabilities in real time. Numerous problems, including bleeding and infection, may arise from the invasive procedures and specialized monitoring equipment needed to quantify dynamic parameters such as the stroke volume variation (SVV), volume variability index (PVI), and pulse pressure variation (PPV) [15,16]. This technology helps minimize the dangers of invasive procedures and specialized monitoring equipment. The inferior vena cava (IVC), which is the largest vein in the human body, is linked to right atrial pressure and blood volume. Consequently, the IVC serves as a crucial focal point during ultrasound evaluations of hemodynamics [17–18]. The IVC index obtained through ultrasound serves as a measure of the efficiency of systemic venous return to the heart. The primary purpose of this index to assess the correlation between venous return volume and cardiac function.

Multiple studies have investigated the prognostic importance of changes in the IVC diameter (∆IVC) for fluid reactivity in hospitalized patients in critical condition. Long et al. [19] found that (∆IVC) had limited predictive ability for fluid reactivity among critically ill patients who were breathing spontaneously. According to the study conducted by Kim et al. [20], alterations in the diameter of the IVC provide reliable diagnostic precision for forecasting liquid reactivity in spontaneously breathing critically ill individuals. Si et al. [21] proposed that ∆IVC had improved diagnostic accuracy among mechanically ventilated patients when the tidal volume (TV) was ≥ 8 ml/kg compared to a TV ≤ 8 ml/kg. Alvarado et al. [22] reported that ∆IVC had good predictive performance for fluid responsiveness in critically ill adult patients on mechanical ventilation with a TV ≤ 8 ml/kg and without dyspnea or arrhythmia. However, there are still inconsistencies in terms of differentiating between spontaneously breathing and mechanically ventilated septic patients. For mechanically ventilated patients, ∆ IVC was referred to as inferior vena cava distensibility (dIVC), and for spontaneously breathing patients, it was referred to as inferior vena cava collapsibility (cIVC). This study aimed to consolidate the literature on the predictive accuracy of changes in IVC diameter for liquid reactivity among septic individuals, thus highlighting the importance of the IVC in managing volume in sepsis patients.

## Materials and methods

This systematic review and meta-analysis carried out was conducted in accordance with the Preferred Reporting Items for Systematic Reviews and Meta-Analysis of Diagnostic Test Accuracy (PRISMA-DTA) statement [23]. The study protocol was formally registered in PROSPERO with the registration number CRD42023469308 to maintain transparency and reliability.

### Selection criteria

(1) The inclusion criteria included studies published in Chinese and English that focused on patients with sepsis or septic shock. The research subjects in the original literature should identify whether they are mechanically ventilated or spontaneously breathing patients. Mechanically ventilated patients should collect ventilator parameters.
(2) The diagnostic method involved measuring patients’ ∆ IVC via ultrasound. ∆ IVC was also referred to as dIVC and cIVC. The purpose of this study was to assess the predictive accuracy of ultrasonographic measurements of ∆ IVC for fluid responsiveness in patients with sepsis, utilizing measures such as sensitivity, specificity, true positives (TPs), true negatives (TNs), false positives (FPs), and false negatives (FNs).
(3) The exclusion criteria were as follows: expectant females, individuals younger than 18 years of age, duplicate publications, studies lacking data for a 2x2 chart, review articles, conference presentations, case studies, and animal research.

### Search strategy

**Fig 1:**
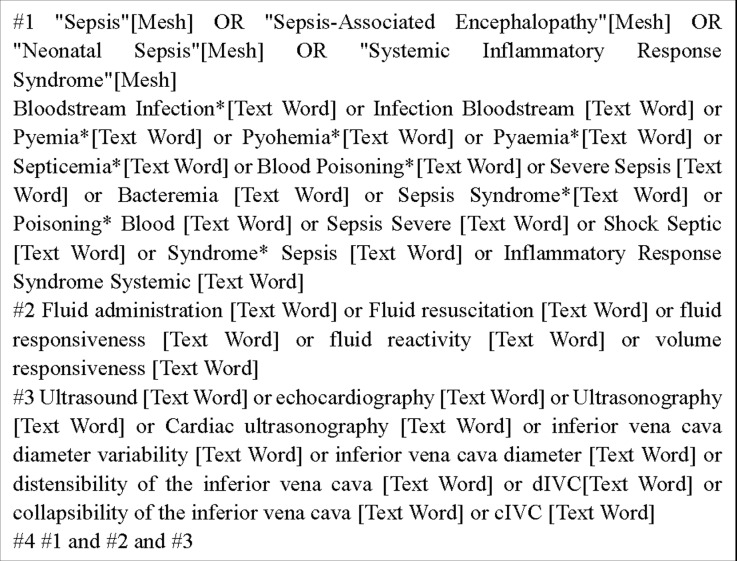
Search strategy of PubMed database.

The Embase, Web of Science, Cochrane Library, MEDLINE, PubMed, Wanfang, CNKI, CBM and VIP databases were comprehensively searched from inception to February 29, 2024. The search strategy was as follows: (sepsis) OR (sepsis-associated encephalopathy) OR (systemic inflammatory response syndrome) OR (neonatal sepsis) OR (bloodstream infection) OR (infection bloodstream)) AND (inferior vena cava diameter) OR (IVC) OR (inferior vena cava distensibility index) OR (inferior vena cava collapsibility index) OR (inferior vena cava respiratory variation index) OR (dIVC) OR (cIVC) OR (IVC-RVI) OR (caval index)) AND (fluid administration) OR (fluid resuscitation) OR (fluid responsiveness) OR (fluid reactivity) OR (volume responsiveness)). PubMed database which showed in Fig 1.

### Study selection

During the first round of the study selection process, two independent reviewers examined the titles and abstracts of the citations, extracted the data and performed cross-verification. Two researchers conducted the screening and data extraction on the basis of the literature, and any disagreements were resolved by discussion with a third researcher. Endnote X9 was utilized to manage the literature. The initial screening involved reviewing the article title, followed by assessing the abstract and full text to determine eligibility, excluding any obvious inconsistencies. The following data were extracted: the first author; the source of publication; the date of publication; the duration of the study; the type of study conducted; demographic information about the study population; sample size; sensitivity; specificity; TP, FP, TN, and FN rates; diagnostic methods for fluid reactions; and diagnostic thresholds.

### Assessment of quality

Two separate researchers evaluated the risk of bias in the included literature. Any disagreements were resolved by discussion or by consulting a third party. The quality of the literature was evaluated using the QUADAS-2 tool [24], which assesses aspects such as bias risk and clinical relevance. This tool consists of three tiers and 14 sections.

### Statistical analysis

We conducted the statistical analysis via Stata 15.0 software and Meta-DiSc 1.4. In studies demonstrating heterogeneity, Meta-DiSc 1.4 was utilized to examine threshold effects. We performed Spearman’s correlation analysis and merged effect size indicators in cases without a threshold effect. Meta-analysis was performed using Stata 15.0, which yielded the sensitivity, specificity, summary receiver operating characteristic curve (AUC), positive likelihood ratio (PLR), diagnostic odds ratio (DOR), negative likelihood ratio (NLR), and a forest plot. The evaluation of publication bias was conducted through the utilization of Deeks’ funnel plot. Fagan plots were used to analyze the clinical importance of IVC respiratory variability in predicting liquid reactivity. For the subgroup analysis, we first compared mechanically ventilated and nonmechanically ventilated individuals. We analyzed various ventilator parameters including TV, PEEP, and threshold, which are mechanically ventilated.

## Results

### Search results

**Fig 2:**
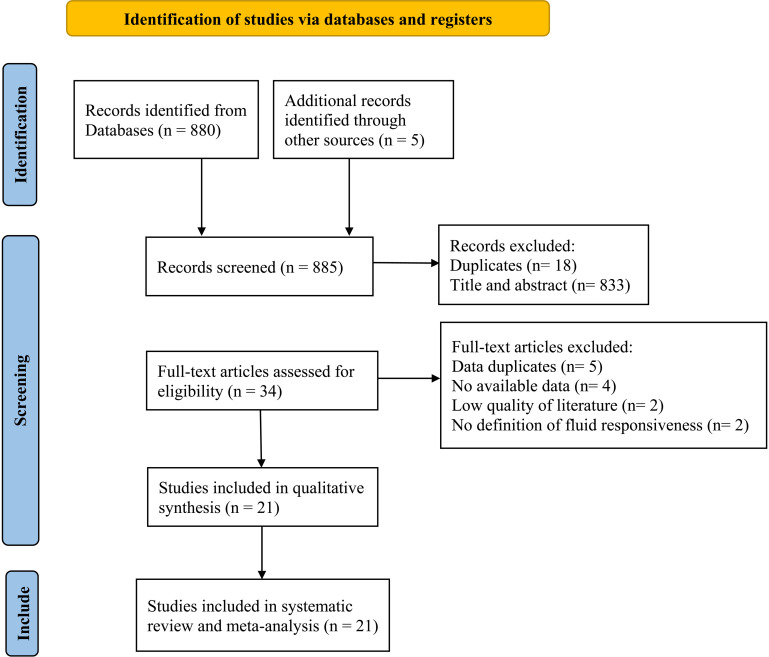
Flowchart of study selection.

A total of 885 papers were initially identified across nine databases. By removing 18 duplicates with the help of the Endnote X9 tool and then excluding 833 more through screening, the full texts of the remaining 34 citations were thoroughly reviewed. Ultimately, 21 research studies were deemed suitable for inclusion, including 14 studies published in English and 7 studies published in Chinese [25–45]. (Fig 2).

### Characteristics of the included studies

A total of 21 articles encompassing 1207 patients with sepsis were reviewed. Of these, 15 articles focused on dIVC, whereas six focused on cIVC. (Tables 1 and 2).

**Table 1:** Main characteristics of the eligible studies.

Author	Year	Country	Device	Measure Site	Fluid challenge	Reference Standard	vasopressors	MV	MV setting	Index Test	Study period	Threshold	Sample Size
Shen	2019	China	US (CX50, Holland)	2 cm from right atrium	500ml NS/LR over 20min	CO > 10%	yes	yes	/	dIVC	2017.05 ~ 2018.10	16.5%	50
Gao	2021	China	US (Viv-id E9,USA)	M-model,2 cm the hepatic vein joins the inferior vena cava	7 ml/kg of LR over 20min	CI ≥ 15%	yes	yes	VT = 6 ~ 10ml/kg	dIVC	2019.01 ~ 2020.02	17.65%	27
Yao	2020	China	US (Sonosite,USA)	M-model, 2 cm from right atrium	500 mL 6% hydroxyethyl starch over 30min	CI ＞ 15%	yes	yes	VT = 10ml/kg	dIVC	2018.10 ~ 2019.10	28%	70
Zhang	2022	China	US (Resona7,China)	/	/	CI ≥ 15%	/	yes	/	dIVC	2020.01 ~ 2021.12	15.7%	110
Chen	2023	China	US (vivid E9,USA)	/	/	CI > 10%	yes	yes	/	dIVC	2018.01 ~ 2020.12	11.77%	98
Zhu	2016	China	US (Sonosite,USA)	M-model, 2 cm from right atrium	500 mL of 6% hydroxyethyl starch/NS over 30min	SV ≥ 15%	yes	yes	/	dIVC	2013.06 ~ 2015.08	19.25%	58
Lu	2018	China	/	2 cm from right atrium	200ml NS over 10min	CI ≥ 10%	yes	yes	/	dIVC	2016.01 ~ 2017.12	20.5%	65
Orhan	2022	Turkey	US (Esaote,Italy)	M-model, 2 cm from right atrium	10 mL/kg of crystalline solution baseline and 15min	CO ≥ 15%	no	yes	VT = 8ml/kg,PPEP = 5 cmH_2_O	dIVC	2018.06.26 ~ 2019.07.01	17.52	40
Charbonn	2014	France	US (Suresnes,France)	M-model, Directly above the hepatic vein junction	7 ml/kg of 6% hydroxyethyl starch 15 min	CI ≥ 15%	yes	yes	VT = 8 ~ 10ml/kg	dIVC	/	21	44
Lu	2017	China	US (Sonosite, USA)	M-model, 2 cm from right	200 mL NS over 10min	CI ≥ 10%	yes	yes	VT = 8 ~ 10ml/kg, PEEP = 5 ~ 12 cmH_2_O	dIVC	2012.01 ~ 2015.12	20.5	49
Theerawit	2016	Thailand	US (SonoSite,USA)	M-model, 2 cm from right atrium	1000 ml NS/500 ml 6% hydroxyethyl starch/5% human albumin over 30min	CO ≥ 15%	yes	yes	VT ≥ 8ml/kg PEEP = 8 ~ 10 cmH_2_O	dIVC	2012.11 ~ 20.13.12	10.2	29
Saber	2022	Egypt	/	M-model, 2 cm from right atrium	500mL NS 10min, Two measurements。	CO ≥ 15%	yes	yes	VT = 6 ~ 8ml/kg, PEEP = 0 ~ 5 cmH_2_O	dIVC	2017.10 ~ 2018.10	14.5	40
Feissel	2004	France	/	M-model, 3 cm from right atrium	8ml/kg 6% hydroxyethyl starch 20min	CO ≥ 15%	/	yes	VT ≥ 8 ~ 10 mL/kg	dIVC	/	12	39
Abdelma	2023	Egypt	US(GE HealthCare Vivid,USA)	/	30ml/kg of crystalline solution	CO ≥ 15%	yes	yes	VT = 8ml/kg,PEEP = 5 cmH_2_O	dIVC	2021.09 ~ 2022.03	13.3	34
He	2023	China	US(Zonare,USA)	the IVC under the raphe	200 mL NS over 10min	SV ≥ 15%	/	yes	PEEP = 4 cmH_2_O	dIVC	2018.04 ~ 2021.02	16.5	102
Preau	2017	France	US(vivid-i/Vivid-S5,USA)	1.5 to 2 cm Hepatic vein junction with IVC/3–4 cm from right atrium	500ml 4% Gelatin over 30min	SV ≥ 15%	yes	no	/	cIVC	2011.11 ~ 2014.01	48	90
Zhao	2016	China		2 cm Hepatic vein junction with IVC	500 ml 6% hydroxyethyl starch 30min	CI ≥ 15%	yes	no	/	cIVC	2013.10 ~ 2014.03	12.9	42
Caplan	2020	France	US(Vivid-i/Vivid-S5,USA)	4 cm from right atrium	500ml 4% Gelatin over 30min	SV ≥ 10%	yes/NE	no	/	cIVC	2011.11 ~ 2015.05	44	81
Elsaeed	2022	Egypt	US(GE,USA)	M model lower edge of the ribcage	7 ml/kg of LR over 30min	CI ≥ 15%	no	no	/	cIVC	/	35	40
Murat	2016	Turkey	US(Philips EPIQ 5,USA)	M model, 0.5 to 3 cm from right atrium-hepatic vein junction	PLR	CI ≥ 15%	/	no	/	cIVC	/	35	44
Perrine	2018	France	US(vivid-i/Vivid-S5,USA)	1.5 to 2 cm Hepatic vein junction with IVC/3–4 cm from right atrium	500ml 4% Gelatin over 30min	Velocity time integral≥10%	yes/NE	no	/	cIVC	2012.05 ~ 2015.05	39	55

Ultrasonographic = US, inferior vena cava = IVC, inferior vena cava diameter = ∆IVC, inferior vena cava distensibility = dIVC, inferior vena cava collapsibility = cIVC, MV = mechanical ventilation; PEEP = positive end expiratory pressure, tidal volume = VT, NS = Normal saline, PLR = passive leg raising, CO = cardiac output, CI = cardiac index, SV = stroke volume,/ = not reported, NE = Norepinephrine.

**Table 2:** 2-by-2 table of the eligible studies.

Author	Year	TP	FP	FN	TN	Total	Sensitivity	Specificity	AUC
Shen	2019	20	7	5	18	50	80%	72%	0.777
Gao	2021	19	1	0	7	27	100.00%	87.50%	0.924
Yao	2020	34	2	5	29	70	87.20%	93.10%	0.94
Zhang	2022	44	7	22	37	110	66.00%	85.00%	0.709
Chen	2023	53	28	3	14	98	95.08%	34.14%	0.664
Zhu	2016	17	3	15	23	58	53.10%	88.50%	0.733
Lu	2018	19	4	12	30	65	60.30%	89.70%	0.826
Orhan	2022	18	5	5	12	40	77.50%	72.50%	0.833
Charbonn	2014	10	7	16	11	44	38%	61%	0.43
Lu	2017	18	5	9	17	49	67%	77%	0.805
Theerawit	2016	12	3	4	10	29	75%	76.90%	0.688
Saber	2022	17	2	3	18	40	85%	90%	0.913
Feissel	2004	15	2	1	21	39	93%	92%	/
Abdelma	2023	21	0	2	11	34	91.30%	100%	0.925
He	2023	35	7	19	41	102	65.4	84.9	0.858
Preau	2017	42	4	8	36	90	84%	90%	0.89
Zhao	2016	32	0	0	10	42	100%	100%	0.917
Caplan	2020	38	1	3	39	81	93%	98%	0.98
Elsaeed	2022	23	1	1	15	40	95.8	93.7	0.97
Murat	2016	18	3	5	18	44	78%	85%	0.825
Perrine	2018	27	3	2	23	55	93%	88%	0.93

true positives = TP, false positives = FP, false negatives = FN, true negatives = TN, area under the receiver operating characteristic curve = AUC.

### Assessment of quality

**Fig 3:**
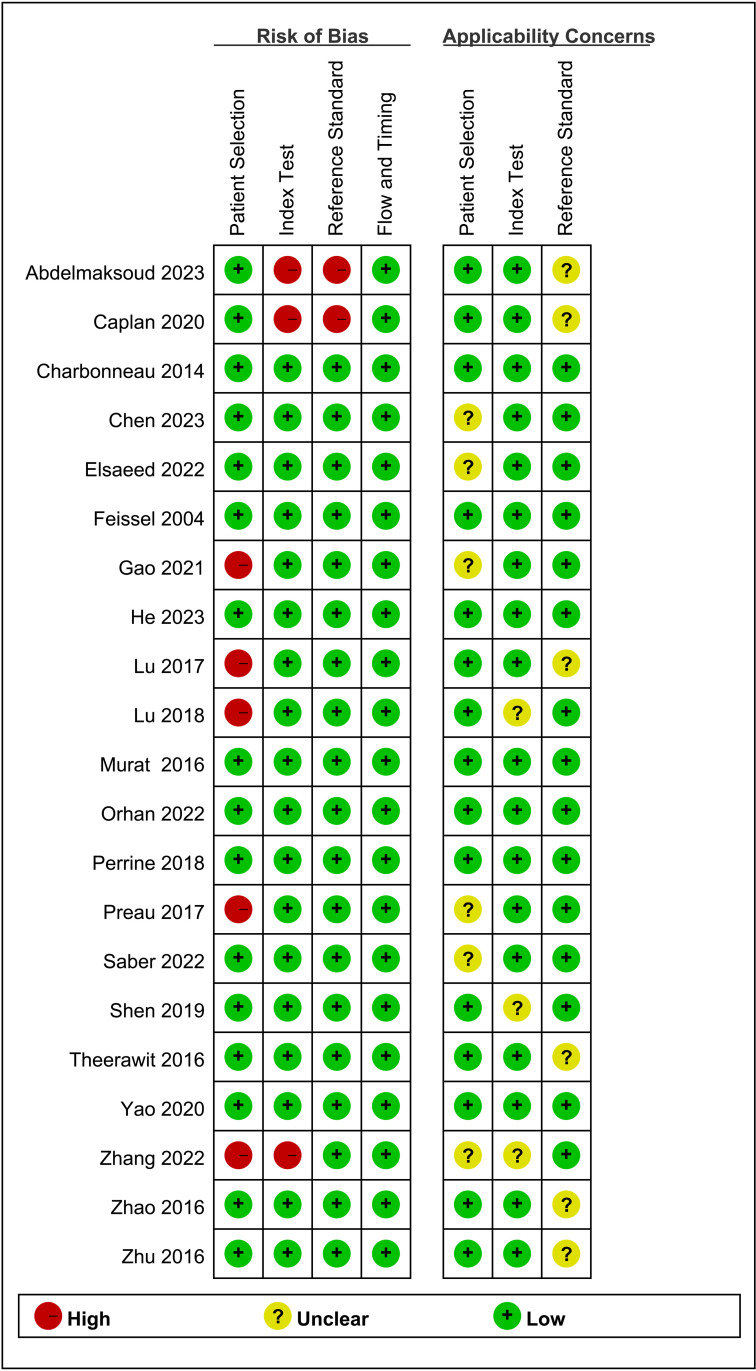
Quality assessment of diagnostic accuracy studies criteria for the included studies.

**Fig 4:**
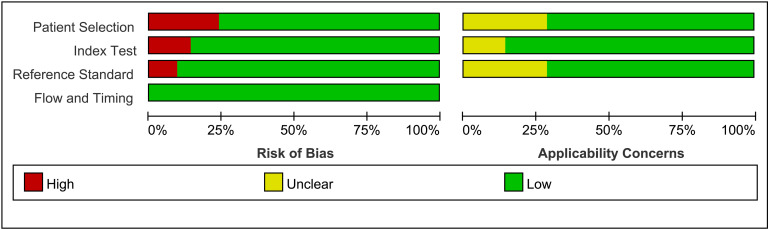
Quality assessment of diagnostic accuracy studies criteria for the included studies.

Using Review Manager 5.3 software, the quality of all included studies was assessed using the QUADAS-2 tool. The findings indicated that the literature was generally high-quality, with potential biases regarding patient selection, the index test, and the reference standard (Figs 3 and 4).

### Statistical analysis

#### Threshold effect test.

The threshold effect test was performed using Meta-DiSc 1.4 software. The results revealed Spearman correlation coefficients of -0.440 (P = 0.046) for ∆ IVC, -0.157 (P = 0.576) for dIVC, and -0.543 (P = 0.266) for cIVC. These findings suggest a potential threshold effect on ∆ IVC, whereas no threshold effect was observed on dIVC and cIVC, allowing their effect sizes to be combined.

#### Meta-analysis.

The sensitivity and specificity of ∆ IVC were 0.84 (95% CI 0.76, 0.90) and 0.87 (95% CI 0.80, 0.91), respectively. The sensitivity and specificity of dIVC were 0.79 and 0.82, respectively. The sensitivity and specificity of cIVC were 0.92 and 0.93, respectively. The results for the other indicators are presented in detail in Table 3. The forest plots depicting the results of the meta-analysis are provided in the appendix of the manuscript (Figs 5-16).

**Table 3:** ∆ IVC, dIVC and cIVC of meta-analysis.

Group	sensitivity	specificity	PLR	NLR	DOR	AUC
∆IVC	0.84	0.87	6.26	0.18	34.03	0.92
	0.76 ~ 0.90	0.80 ~ 0.91	3.93 ~ 9.97	0.12 ~ 0.29	15.09 ~ 76.76	0.89 ~ 0.94
dIVC	0.79	0.82	4.42	0.26	17.10	0.88
	0.68 ~ 0.86	0.73 ~ 0.89	2.83 ~ 6.92	0.17 ~ 0.40	8.10 ~ 36.09	0.84 ~ 0.90
cIVC	0.92	0.93	13.52	0.09	156.62	0.97
	0.83 ~ 0.96	0.86 ~ 0.97	6.16 ~ 29.66	0.04 ~ 0.19	40.11 ~ 611.56	0.95 ~ 0.98

positive likelihood ratio = PLR, negative likelihood ratio = NLR, diagnostic odds ratio = DOR, area under the receiver operating characteristic curve = AUC.

**Fig 5:**
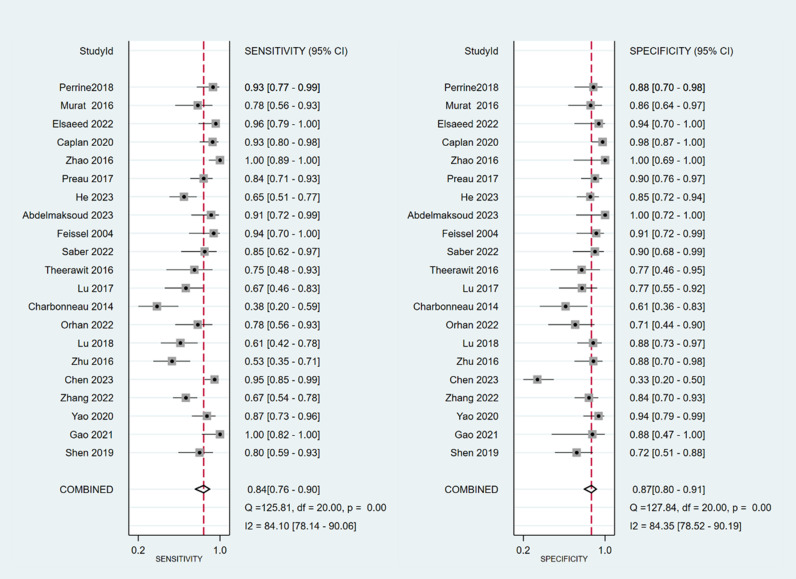
Forest plot of sensitivity and specificity in the diagnosis of ∆ IVC.

**Fig 6:**
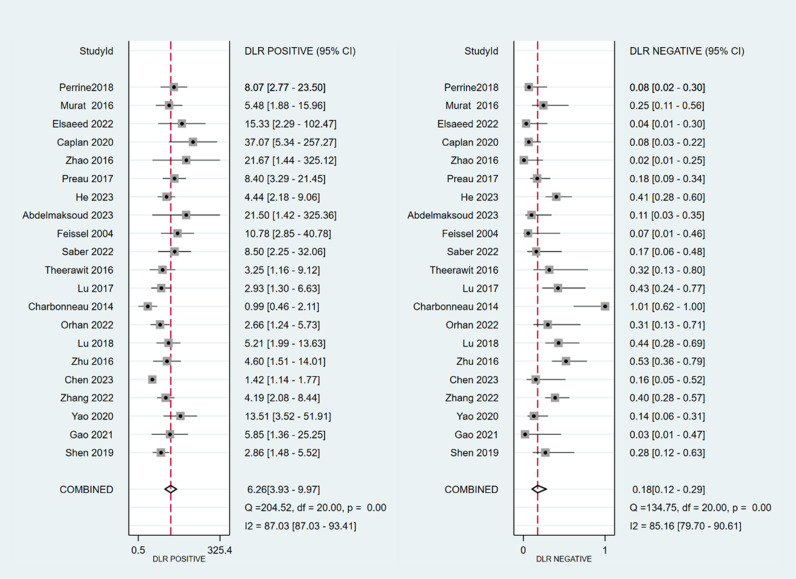
Forest plot of PLR and NLR in the diagnosis of ∆ IVC.

**Fig 7:**
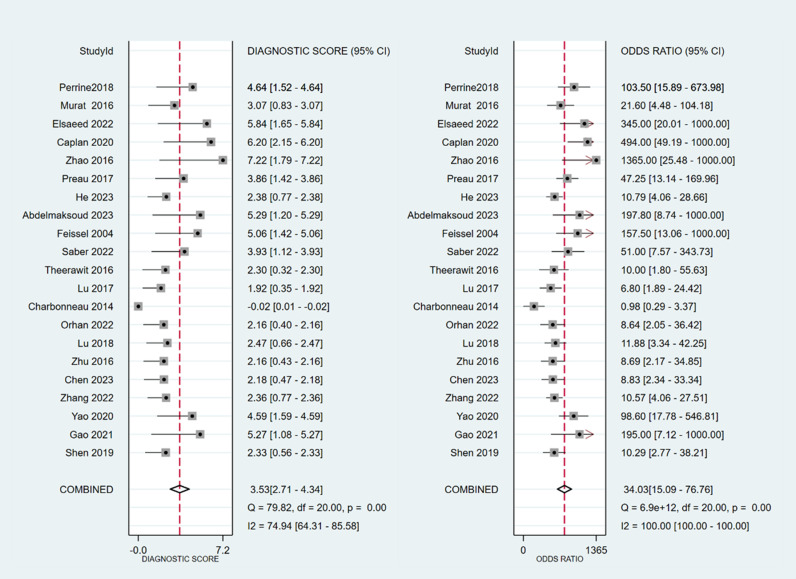
Forest plot of DOR in the diagnosis of ∆ IVC.

**Fig 8:**
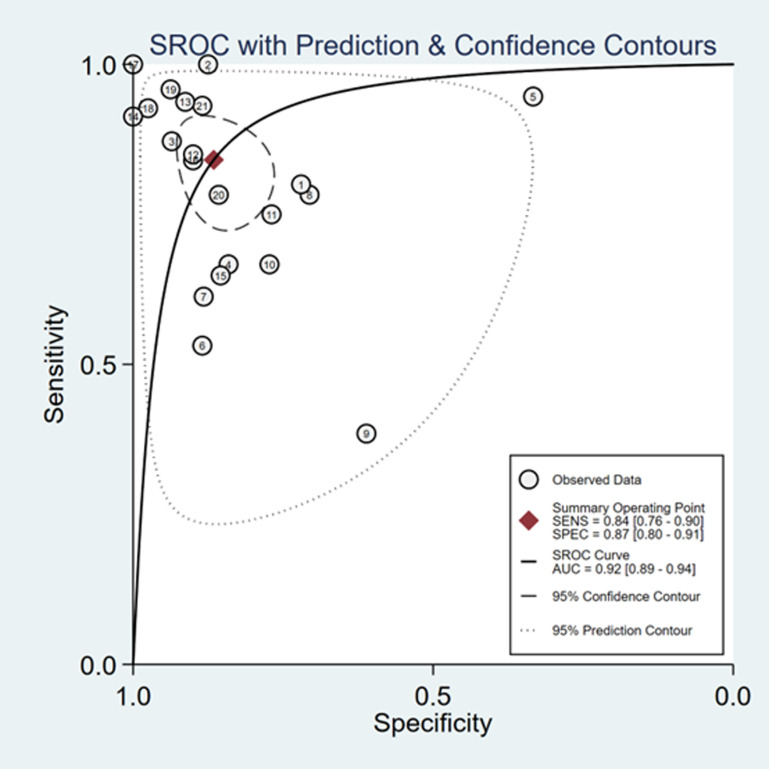
Summary receiver operator characteristic (SROC) curve in the prediction of ∆ IVC. SENS = sensitivity, SPEC = specificity, AUC = area under the receiver operating characteristic curve.

**Fig 9:**
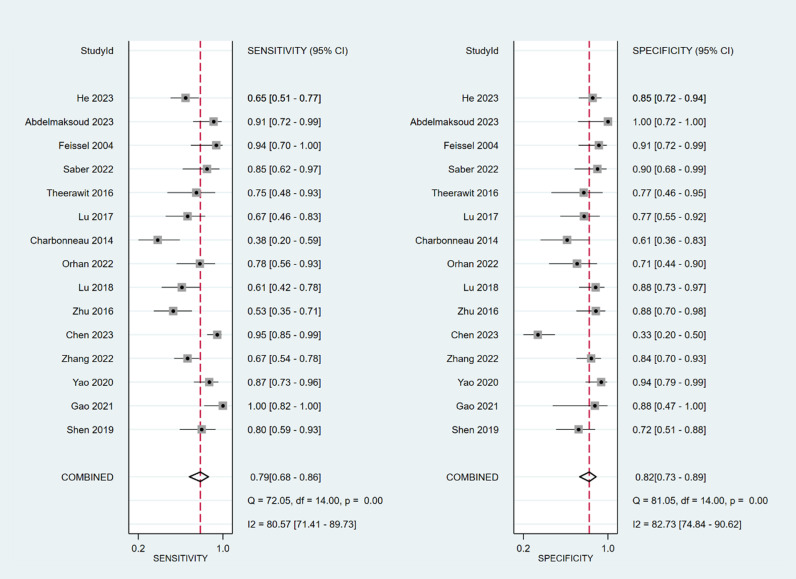
Forest plot of sensitivity and specificity in the diagnosis of dIVC.

**Fig 10:**
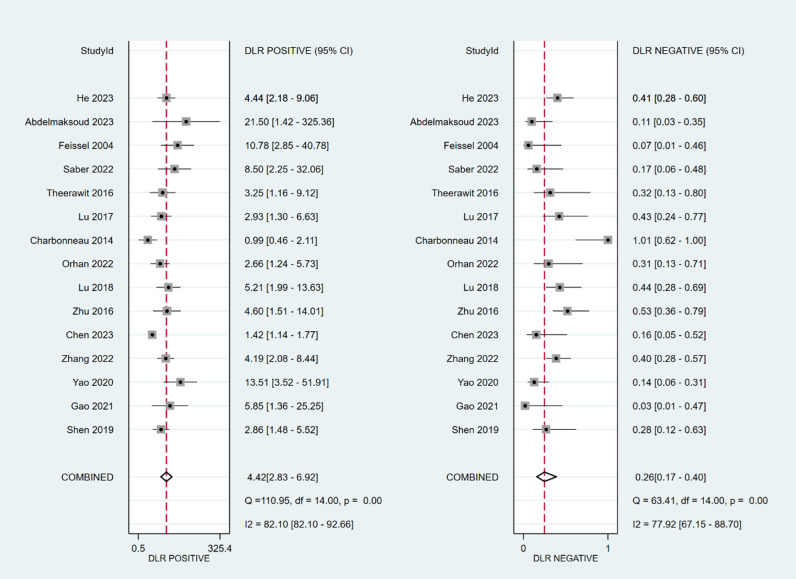
Forest plot of PLR and NLR in the diagnosis of dIVC.

**Fig 11:**
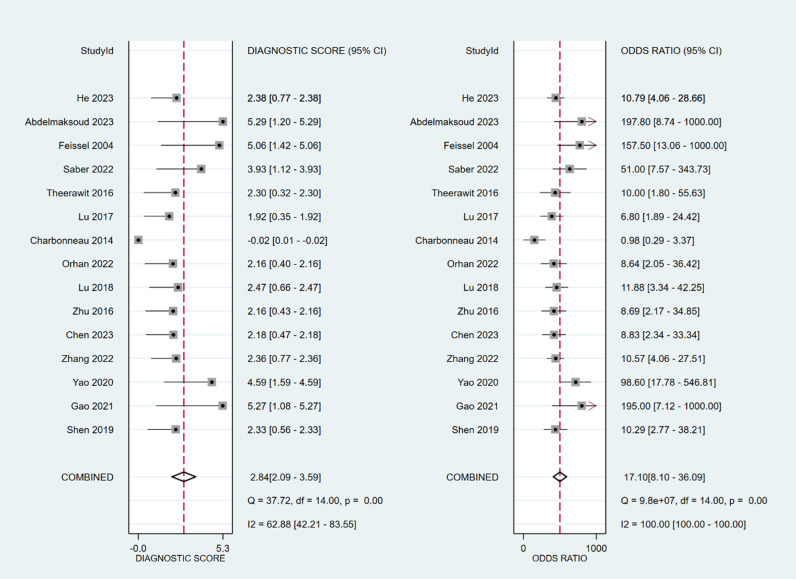
Forest plot of DOR in the diagnosis of dIVC.

**Fig 12:**
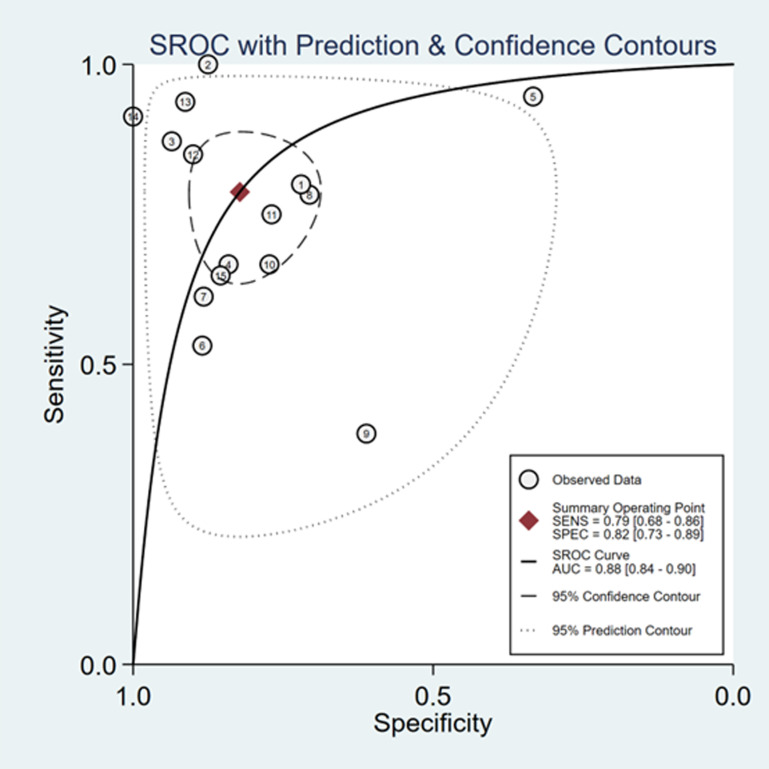
Summary receiver operator characteristic (SROC) curve in the prediction of dIVC. SENS = sensitivity, SPEC = specificity, AUC = area under the receiver operating characteristic curve.

**Fig 13:**
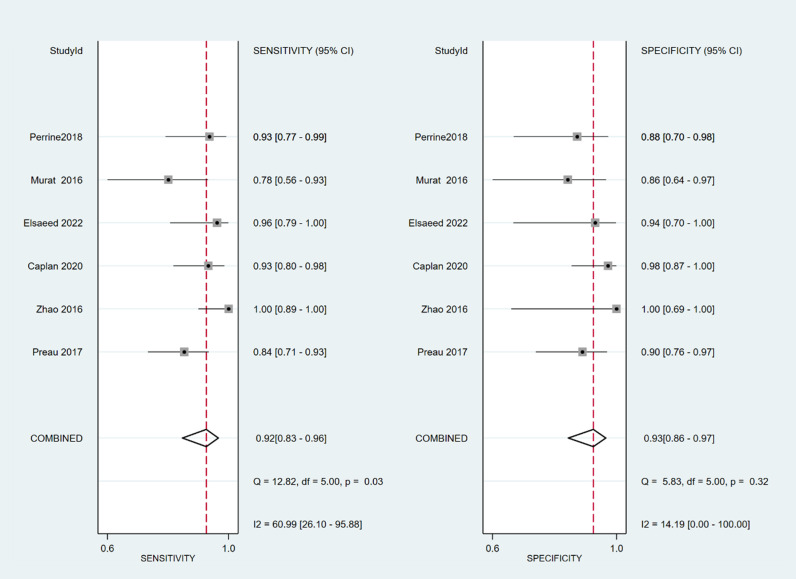
Forest plot of sensitivity and specificity in the diagnosis of cIVC.

**Fig 14:**
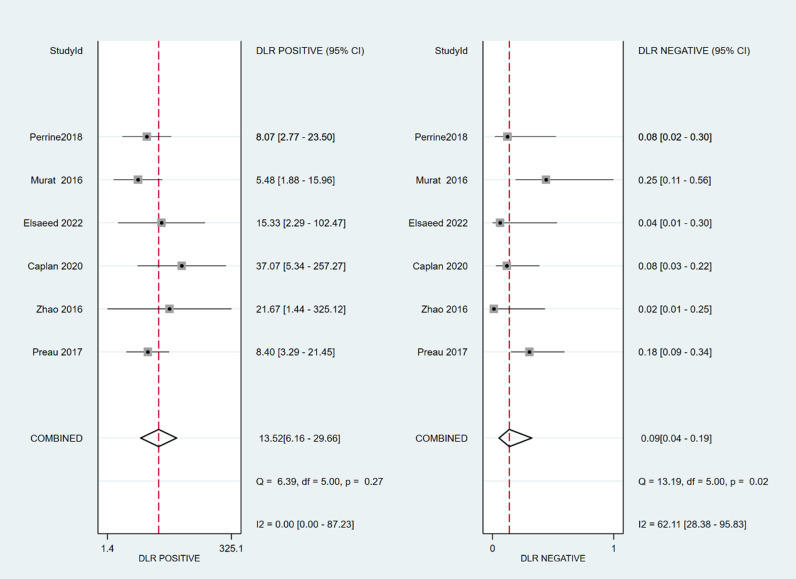
Forest plot of PLR and NLR in the diagnosis of cIVC.

**Fig 15:**
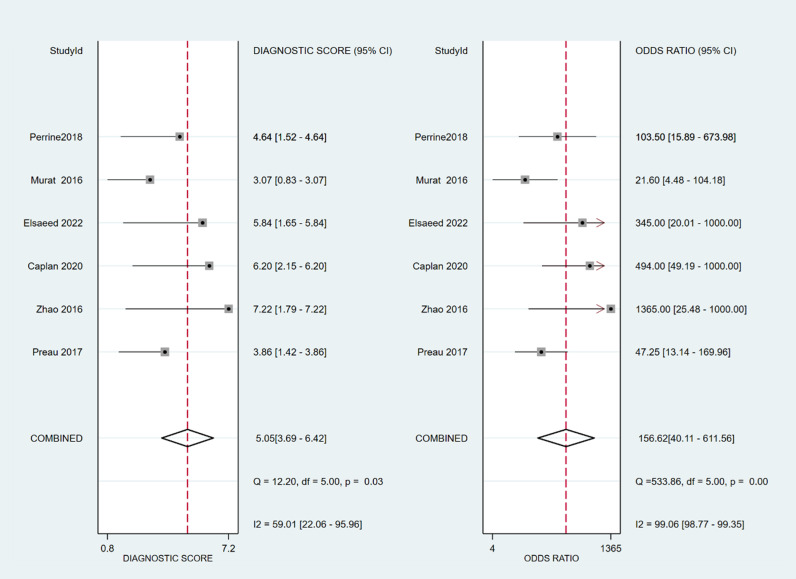
Forest plot of DOR in the diagnosis of cIVC.

In Deeks’ funnel plots, the values were 0.28, 0.23, and 0.60, indicating that the included studies had no publication bias (Figs 17-19). The 50% predictive probability results were 86%, 82%, and 93%, and the negative posttest probabilities were 16%, 21%, and 8%, respectively (Figs 20-22).

**Fig 16:**
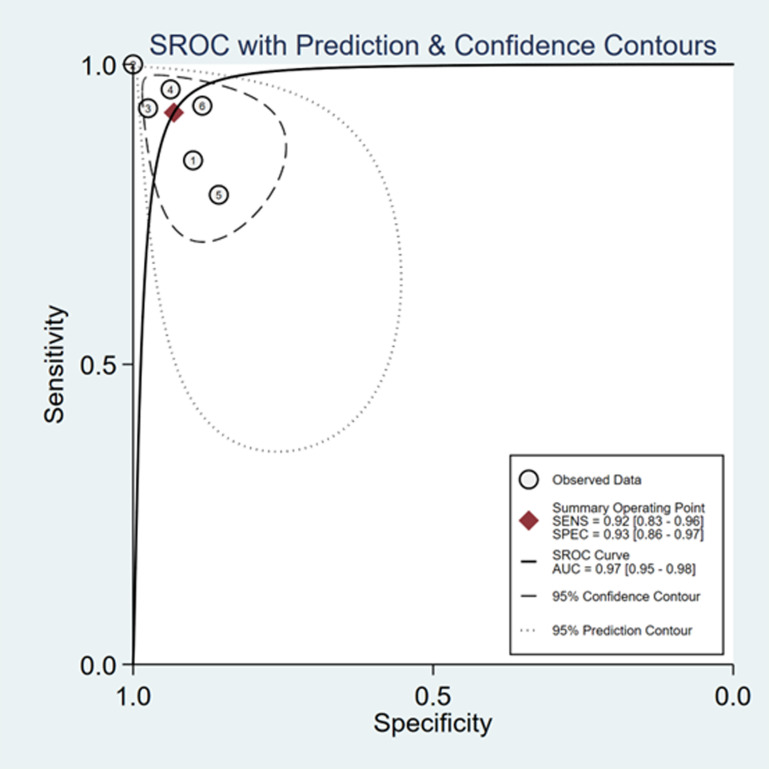
Summary receiver operator characteristic (SROC) curve in the prediction of cIVC. SENS = sensitivity, SPEC = specificity, AUC = area under the receiver operating characteristic curve.

**Fig 17:**
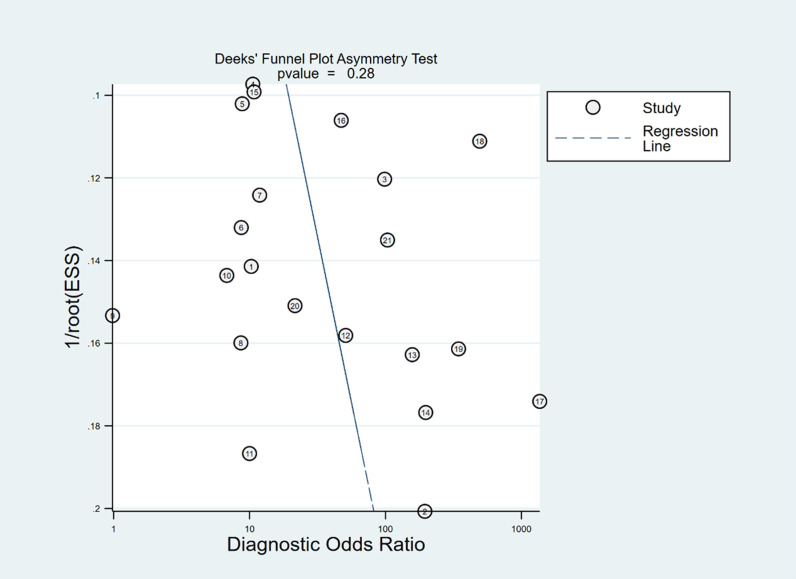
Deek’s funnel plots of ∆ IVC.

**Fig 18:**
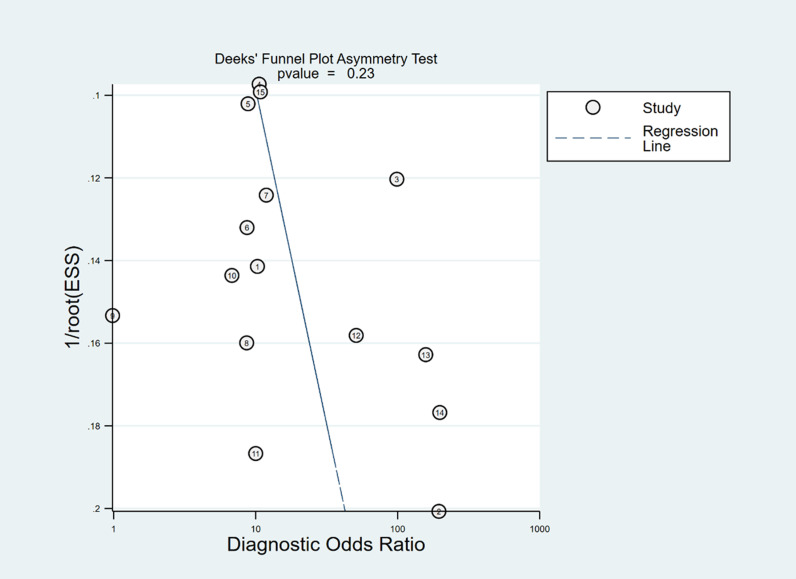
Deek’s funnel plots of dIVC.

**Fig 19:**
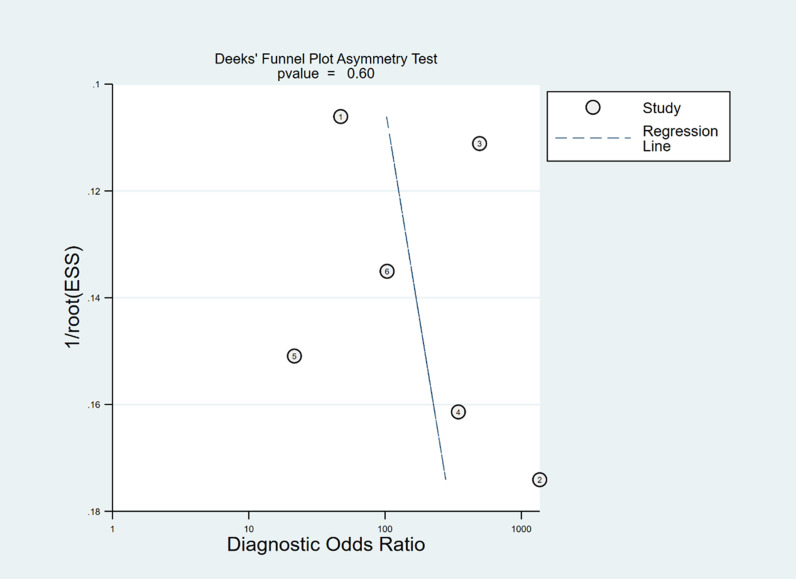
Deek’s funnel plots of cIVC.

**Fig 20:**
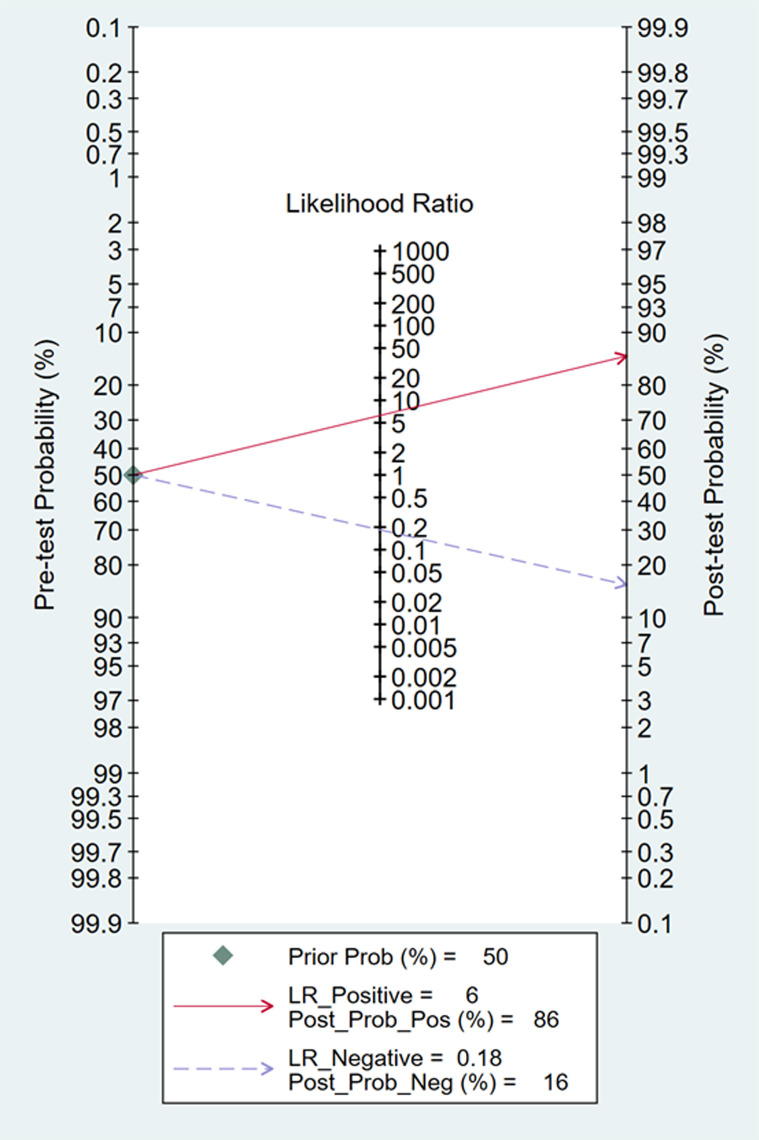
Fangan plots of ∆ IVC.

**Fig 21:**
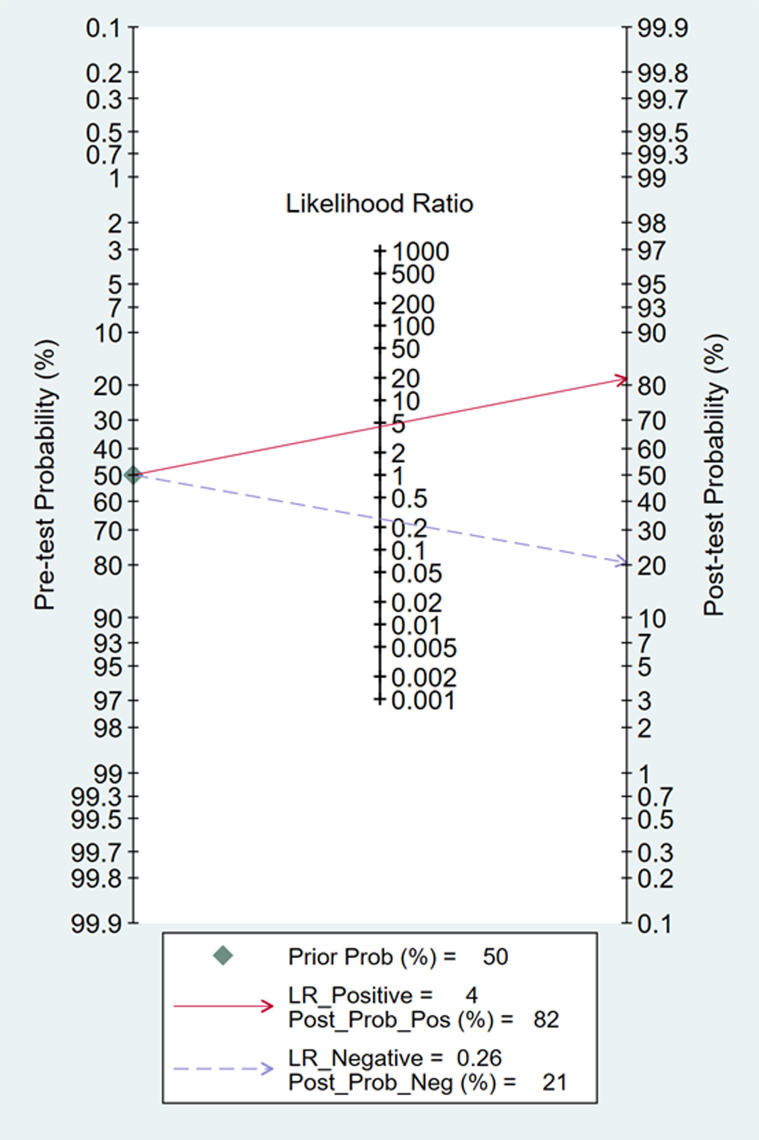
Fangan plots of dIVC.

**Fig 22:**
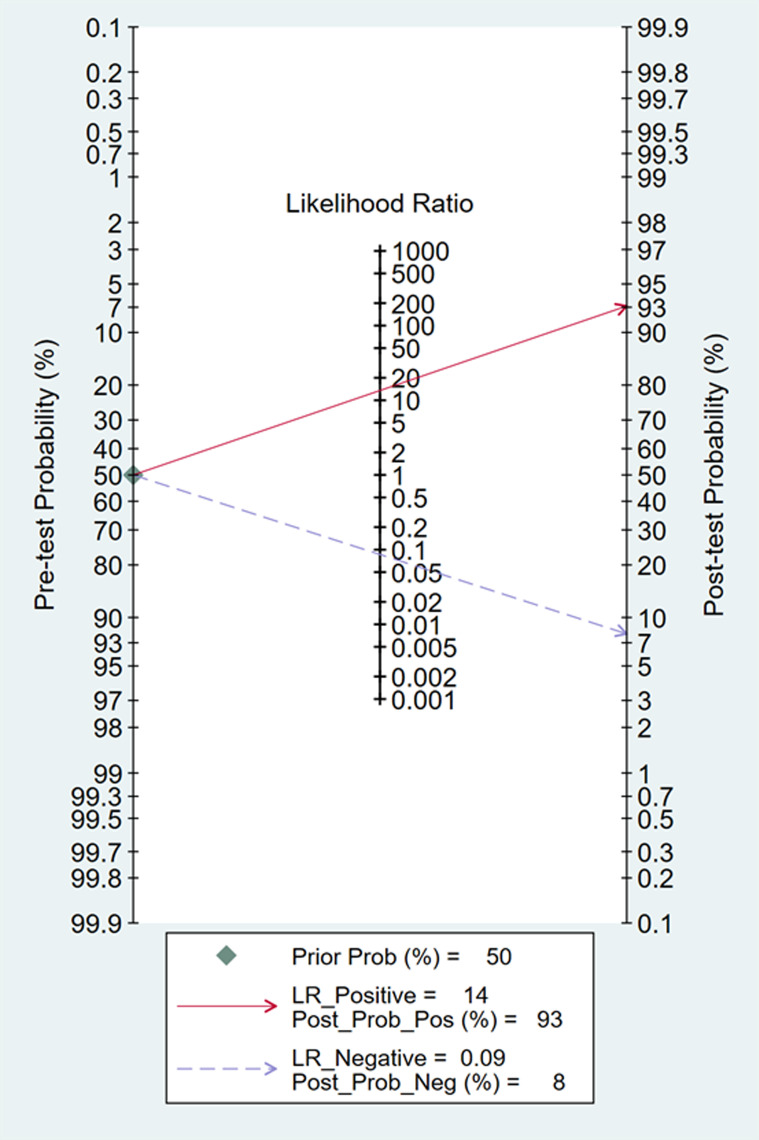
Fangan plots of cIVC. Subgroup analysis.

Currently, there is no evidence that a single evaluation parameter or index can be utilized as an endpoint on its own to direct fluid resuscitation in sepsis patients. Our subgroup analysis is based on previous systematic review results, basic ventilator parameters (TV = 6-8mL/kg, PEEP = 5 cmH2O), and dose selection for fluid resuscitation. Each enrolled patient with septic shock was categorized into the low-volume (200ml), medium-volume (7 mL/kg fluid), or high-volume (above 500 mL) fluid group according to the infusion volume. We conducted subgroup analyses based on TV, PEEP, infusion volume, and diagnostic threshold. A meta-regression analysis was conducted on ∆ IVC in the dIVC and cIVC, revealing a statistically significant difference of SE = 2.022 (95% CI: 1.81–31.48, P = 0.0081), which could account for the observed heterogeneity. Subgroup analyses were then performed based on TV(P = 0.580), PEEP(P = 0.732), infusion volume(P = 0.417), and diagnostic threshold (P = 0.472) in the dIVC, as well as infusion volume(P = 0.791) and diagnostic threshold (P = 0.846) in the cIVC. The analyses did not yield significant results (Tables 4–5).

**Table 4:** dIVC of Subgroup analyses.

Subgroup	sensitivity	specificity	PLR	NLR	DOR	AUC	Meta-regression (*P* value)
VT(ml/kg)							0.580
≥8	0.75	0.82	3.92	0.28	16.30	0.885	
	0.68 ~ 0.82	0.75 ~ 0.88	1.81 ~ 8.50	0.13 ~ 0.58	4 ~ 66.49	0.816	
≤8	0.85	0.85	5.61	0.20	30.96	0.888	
	0.74 ~ 0.92	0.74 ~ 0.92	1.58 ~ 19.85	0.11 ~ 0.36	5.52 ~ 173.68	0.819	
PEEP(cmH_2_O)							0.732
PEEP≤5	0.76	0.85	4.49	0.26	18.67	0.871	
	0.67 ~ 0.83	0.77 ~ 0.92	2.36 ~ 8.54	0.14 ~ 0.47	6.36 ~ 54.81	0.802	
PEEP≥5	0.78	0.79	3.07	0.30	10.79	0.819	
	0.67 ~ 0.86	0.67 ~ 0.89	1.90 ~ 4.99	0.18 ~ 0.50	4.10 ~ 28.38	0.753	
Infusion volume							0.417
500ml	0.780	0.870	5.130	0.240	23.230	0.895	
	0.71 ~ 0.84	0.79 ~ 0.92	2.75 ~ 9.57	0.13 ~ 0.45	8.84 ~ 61.7	0.826	
200ml	0.64	0.85	4.02	0.42	9.78	0.753	
	0.55 ~ 0.73	0.76 ~ 0.91	2.52 ~ 6.43	0.33 ~ 0.55	5.05 ~ 18.97	0.696	
≥7ml/kg	0.740	0.770	3.190	0.220	16.780	0.806	
	0.63 ~ 0.83	0.65 ~ 0.87	1.12 ~ 9.08	0.04 ~ 1.08	1.51 ~ 186.11	0.741	
Threshold (%)							0.472
≤16.5	0.79	0.75	4.25	0.27	14.95	0.856	
	0.73 ~ 0.83	0.69 ~ 0.81	1.98 ~ 9.11	0.19 ~ 0.40	8.14 ~ 27.47	0.787	
＞16.5	0.69	0.83	3.53	0.40	10.81	0.872	
	0.62 ~ 0.75	0.76 ~ 0.88	1.87 ~ 6.64	0.24 ~ 0.66	3.51 ~ 33.33	0.802	

positive likelihood ratio = PLR, negative likelihood ratio = NLR, diagnostic odds ratio = DOR, area under the receiver operating characteristic curve = AUC.

**Table 5:** cIVC of Subgroup analyses.

Subgroup	sensitivity	specificity	PLR	NLR	DOR	AUC	Meta-regression (*P* value)
Infusion volume							0.791
500ml	0.89	0.93	9.61	0.14	93.09	0.974	
	0.83 ~ 0.94	0.86 ~ 0.97	4.36 ~ 21.21	0.06 ~ 0.29	19.52 ~ 443.89	0.926	
7ml/kg	0.958	0.937	/	/	/	0.970	
	/	/	/	/	/	0.861 ~ 0.999	
PLR	0.93	0.88	/	/	/	0.930	
	0.77 ~ 0.99	0.69 ~ 0.97	/	/	/	0.86 ~ 0.10	
Threshold (%)							0.846
＞35	0.89	0.92	10.07	0.12	101.73	0.960	
	0.82 ~ 0.94	0.86 ~ 0.97	4.87 ~ 20.80	0.06 ~ 0.23	28.81 ~ 359.19	0.906	
≤35	0.92	0.91	7.91	0.08	138.52	0.960	
	0.84 ~ 0.97	0.80 ~ 0.978	3.28 ~ 19.09	0.01 ~ 0.49	10.72 ~ 1789.77	0.903	

positive likelihood ratio = PLR, negative likelihood ratio = NLR, diagnostic odds ratio = DOR, area under the receiver operating characteristic curve = AUC.

## Discussion

This study analyzed a total of 21 papers involving 1207 septic patients. This meta-analysis is the first study to assess the predictive value of cIVC and dIVC for liquid reactivity among septic individuals who are mechanically ventilated and spontaneously breathing. The findings indicated that ∆IVC had good overall performance in predicting fluid reactivity in septic patients, with a combined sensitivity, specificity, and AUC of 0.84, 0.87, and 0.92, respectively, exceeding the values reported in previous systematic reviews [19,20,46–48].

The subgroup analysis could not determine the effect of vasopressors on fluid resuscitation because only two of the assessed studies included patients who did not receive vasopressors. Vasopressors and fluid resuscitation are essential components of hemodynamic support for treating patients with septic shock [49]. Vasopressors may also be utilized until fluid resuscitation is finished, according to ESICM recommendations [6]. According to previous research, treating patients with septic shock necessitates multiangle assessment, individualized treatment, quantified and targeted fluid management and assessment, and prudent and standardized use of vasopressors, all of which are helpful in the resuscitation of patients experiencing early septic shock [50].

A rational fluid approach necessitates that we view septic shock as a multiphase illness, and numerous indicators or techniques are combined on the basis of the patient’s volume responsiveness. Subgroup analyses by Long [19] et al. and Kim et al. [20]. demonstrated that ∆IVC was an effective predictor of fluid reactivity, with AUCs of 0.85 and 0.87, respectively; however, ∆ IVC did exhibit lower sensitivity. The cIVC showed excellent performance in terms of predicting fluid reactivity in spontaneously breathing septic individuals, displaying superior diagnostic accuracy compared with the dIVC in mechanically ventilated septic patients. This finding is consistent with the study by Orso et al. [47] but contrary to those of Zhang et al. [46], Long et al. [19] and Kim et al. [20], possibly due to the inclusion of a diverse range of critically ill patients. However, Long et al. [19]. and Orso et al. [47]. included both adults and children in their studies. Since children have different physiological characteristics, such as vascular compliance, respiratory rate, and TV, than adults, caution should be taken when comparing and analyzing the results.

The latest edition of the Sepsis and Infectious Shock Guidelines, which was published by the Surviving Sepsis Campaign, recommends the use of dynamic measurements to assess fluid responsiveness [6]. Ultrasound is utilized for hemodynamic evaluation with a focus on volume. The IVC, which is the largest venous trunk in the body, is closely linked to right atrial pressure and blood volume. It is pliant and easily dilated, and its internal diameter and degree of collapse are used to evaluate volume status in critically ill patients [51]. ∆ IVC provides a comprehensive three-dimensional assessment of the internal diameter of the IVC, aligning with current practices. Meta-regression analysis of ∆ IVC among different respiratory tract types revealed significant differences, with dIVC and cIVC potentially contributing to the observed heterogeneity. While ∆IVC was termed dIVC and cIVC in mechanically ventilated and spontaneously breathing patients, respectively, subgroup analyses in related studies revealed differences only in respiratory or disease types, thus lacking a solid scientific basis for volume assessment in septic patients. Studies have suggested that the ∆ IVC performs better in predicting fluid responsiveness in mechanically ventilated patients than in spontaneously breathing patients [19,20,46], possibly because of factors such as hypovolemia or changes in intrathoracic pressure during respiration. dIVC has shown promise in predicting fluid responsiveness in mechanically ventilated septic patients, with higher sensitivity, specificity, and AUC values than those reported in previous studies. This improvement may be attributed to the inclusion of more recent studies in the analysis. This mechanism arises from the varying breathing patterns of patients, leading to distinct trends in the variation in the inner diameter of the inferior vena cava during inspiration [52]. Among individuals who breathe spontaneously, negative pressure is present in the chest at the end of expiration. Fluctuations in intrathoracic pressure can result in a decrease in pressure within the right atrium, thereby increasing the speed of venous return. Conversely, during mechanical ventilation, the intrathoracic pressure increases during the inspiratory phase. This elevation in pressure results in an increase in right atrial pressure, which subsequently obstructs venous return.

The determination of fluid responsiveness via dIVC was more accurate in septic patients with ventilator parameters of TV ≤ 8 ml/kg or PEEP ≤5 cmH_2_O than in those with TV ≥ 8 ml/kg or PEEP ≥5 cmH_2_O. However, meta-regression analysis of TV and PEEP did not reveal any significant moderators, and subgroup analysis did not yield any statistically significant differences. These findings align with a study conducted by Alvarado et al. [22]. The results reported by Si et al. [21] were also consistent with our results with respect to PEEP, but those authors reported opposite results concerning tidal volume. In mechanically ventilated patients, the ventilator controls inspiratory flow, resulting in positive pressure and elevated intrathoracic pressure and leading to changes in the IVC diameter [53]. Studies have shown that the use of low TV ventilation modes in septic patients can improve pulmonary oxygenation and reduce airway pressure, thereby lowering the risk of lung injury. Additionally, larger TV may lead to alveolar overdistension, impair pulmonary blood flow perfusion, and redistribute fluids, ultimately compromising the effectiveness of fluid resuscitation [54]. Research indicates that appropriate PEEP can enhance alveolar ventilation, improve oxygenation, and reduce the incidence of pulmonary edema. However, excessively high PEEP may decrease cardiac return, adversely affecting hemodynamics and increasing fluid demands [55]. It is crucial to optimize respiratory conditions through precise ventilator settings to ensure lung protection while avoiding excessive fluid overload. For example, if chest ultrasound demonstrates adequate cardiac preload but PEEP settings result in insufficient circulation, adjustments to fluid administration may be necessary. However, when a patient experiences excessive circulating volume load, the inner diameter of the inferior vena cava expands, and the amplitude of its movement diminishes. Previous studies have several limitations, such as small sample sizes and a lack of differentiation between spontaneously breathing and mechanically ventilated septic patients. A more comprehensive study on ventilator parameter settings is necessary to validate these results.

In septic patients, ∆ IVC was found to be more effective than the comparison group in terms of predicting fluid responsiveness with a 500-ml infusion volume and a dIVC threshold >16.5%. However, a dIVC threshold >16.5% had a sensitivity of 0.69, which was lower than that of the comparison group. Subgroup analysis of the cIVC threshold did not reveal significant differences in diagnostic efficacy. Meta-regression analysis revealed that none of the moderators were significant, and no statistically significant differences were observed. The studies included dIVC and cIVC thresholds ranging from 10.2 to 28 and 12.9 to 48, respectively. Changes in the chest wall and lung compliance may impact pulse pressure changes but do not affect volume status, potentially altering thresholds and accuracy of assessment [56]. Corl et al. [57] studied 124 spontaneously breathing critically ill patients in the ICU by measuring the IVC diameter before and after 500 ml of fluid rehydration and reported that an IVC dilatation index >25% predicted increased cardiac output postfluid infusion in spontaneously breathing patients. The area under the curve was 0.84. Airapetian et al. [58] reported 97% sensitivity and 31% specificity in predicting fluid response in autonomously breathing patients when the ∆ IVC was > 42%. According to the Frank–Starling curve, only approximately 50% of patients are volume-responsive enough to benefit from aggressive fluid resuscitation [59]. Small-volume liquid infusion inevitably decreases the critical values of C0, CI and other indicators for judging volume responsiveness, which requires researchers to have very skilled ultrasound technology to reduce errors caused by operation and subjective judgment. Using threshold-guided fluid resuscitation to guide fluid resuscitation in patients with sepsis can more effectively increase the patient’s cardiac output and correct and maintain the intracellular and extracellular electrolyte balance [9]. Patients with septic shock who complete 30 mL/kg fluid resuscitation within 1–2 hours can significantly reduce 28-day mortality while improving organ function [60]. Fluid overload on ICU day 1 may lower in-hospital mortality risk but could increase mortality after day 3. This indicates that early adequate fluid resuscitation is beneficial, whereas strict fluid restriction in later phases is critical to avoid excessive overload [61]. A high fluid balance is associated with increased mortality in severe sepsis and septic shock patients, while low-volume resuscitation within the initial 24 hours markedly reduces mortality. This suggests that lower fluid resuscitation thresholds may be safer for certain patient populations. Lauralyn et al. [62] suggest that critically ill patients exhibit significant heterogeneity, rendering standardized therapeutic measures increasingly controversial and challenging both theoretically and practically. Initial fluid resuscitation should adhere to an individualized approach. The four-phase resuscitation strategy requires close coordination and timely adjustments based on the patient’s clinical status. However, ineffective implementation persists due to clinicians’ failure to strictly follow guidelines and insufficient clinical experience.

## Limitations

First, heterogeneity in measurement protocols primarily arises from differences between mechanically ventilated and non-ventilated patient cohorts. Second, critically ill patients demonstrate significant heterogeneity that those with heart failure, kidney disease, or other comorbidities may exhibit marked variations in fluid resuscitation efficacy. This necessitates the integration of dynamic parameters, such as ultrasound-guided assessments combined with pulse pressure variation (PPV) or fluid challenge tests, when evaluating fluid resuscitation in septic patients. Additionally, blood lactate levels should be combined to comprehensively assess resuscitation outcomes. Furthermore, the impact of concomitant vasopressor use during fluid resuscitation on the trial results must be considered in our study.

## Conclusion

The findings demonstrate that selecting parameters with tidal volume ≤8 mL/kg, PEEP ≤5 cmH₂O, and fluid volume ≥500 mL is more effective for accurately assessing fluid resuscitation in mechanically ventilated septic patients. For non-ventilated septic patients, maintaining a fluid volume ≥500 mL remains clinically significant. Overall, ∆ IVC showed strong predictive value for liquid reactivity in septic individuals, with the dIVC and cIVC displaying good and excellent accuracy in predicting fluid reactivity in mechanically ventilated and spontaneously breathing septic individuals.

## Supporting information

S1 Checklist Table for the present systematic review (DOCX)

S2 PRISMA_2020_checklist for the present systematic review. (DOCX)

S3 Supplementary information-1.xlsx. (XLSX)

## References

[1] Rudd KE, Johnson SC, Agesa KM, Shackelford KA, Tsoi D, Kievlan DR, et al. Global, regional, and national sepsis incidence and mortality, 1990-2017: analysis for the Global Burden of Disease Study. Lancet 2020;395(10219): 200-211. 31954465 10.1016/S0140-6736(19)32989-7 PMC6970225

[2] Xie J, Wang H, Kang Y, Zhou L, Liu Z, Qin B, et al. The epidemiology of sepsis in Chinese ICUs: a national cross-sectional survey. Critical Care Medicine. 2019;48(3):e209-e218. 10.1097/CCM.0000000000004155 31804299

[3] Fleischmann C, Scherag A, Adhikari NK, Hartog CS, Tsaganos T, Schlattmann P, et al. Assessment of Global Incidence and Mortality of Hospital-treated Sepsis. Current Estimates and Limitations. Am J Respir Crit Care Med. 2016;193(3):259–272. doi: 10.1164/rccm.201504-0781OC 26414292

[4] Prescott HC, Angus DC. Enhancing recovery from sepsis: A review. JAMA. 2018;319(1):62–75. doi: 10.1001/jama.2017.17687 29297082 PMC5839473

[5] Tiru B, DiNino EK, Orenstein A, Mailloux PT, Pesaturo A, Gupta A, et al. The Economic and Humanistic Burden of Severe Sepsis. Pharmacoeconomics, 2015,33(9):925-937. 25935211 10.1007/s40273-015-0282-y

[6] Evans L, Rhodes A, Alhazzani W, Antonelli M, Coopersmith CM, French C, et al. Surviving sepsis campaign: international guidelines for management of sepsis and septic shock 2021. Intensive Care Med. 2021;47(11):1181-1247. 34599691 10.1007/s00134-021-06506-y PMC8486643

[7] Egi M, Ogura H, Yatabe T, Atagi K, Inoue S, Iba T, et al. The Japanese Clinical Practice Guidelines for Management of Sepsis and Septic Shock 2020 (J-SSCG 2020). J Intensive Care. 2021;9(1):53. doi: 10.1186/s40560-021-00555-7 34433491 PMC8384927

[8] Cao Y, Chai YF, Deng Y, Fang BJ, Liu MH, Lu ZQ, et al. China Guidelines for Emergency Treatment of Sepsis/Septic Shock (2018). Chinese Journal of Critical Care Medicine. 2018;38(9):741-756.

[9] Malbrain MLNG, Van Regenmortel N, Saugel B, De Tavernier BD, Van Gaal P-J, Joannes-Boyau O, et al. Principles of fluid management and stewardship in septic shock: it is time to consider the four D’s and the four phases of fluid therapy. Ann Intensive Care. 2018;8(1):66. doi: 10.1186/s13613-018-0402-x 29789983 PMC5964054

[10] Chaves RCF, Barbas CSV, Queiroz VNF, Serpa Neto A, Deliberato RO, Pereira AJ, et al. Assessment of fluid responsiveness using pulse pressure variation, stroke volume variation, plethysmographic variability index, central venous pressure, and inferior vena cava variation in patients undergoing mechanical ventilation: a systematic review and meta-analysis. Crit Care. 2024;28(1):289. doi: 10.1186/s13054-024-05078-9 39217370 PMC11366151

[11] Ansari BM, Zochios V, Falter F, Klein AA. Physiological controversies and methods used to determine fluid responsiveness: a qualitative systematic review. Anaesthesia. 2016;71(1):94–105. doi: 10.1111/anae.13246 26459299

[12] Perner A, Vieillard-Baron A, Bakker J. Fluid resuscitation in ICU patients: quo vadis? Intensive Care Med. 2015;41(9):1667–9. doi: 10.1007/s00134-015-3900-4 26072659

[13] Malbrain ML, Marik PE, Witters I, Cordemans C, Kirkpatrick AW, Roberts DJ, et al. Fluid overload, deresuscitation, and outcomes in critically ill or injured patients: a systematic review with suggestions for clinical practice. Anesthesiol Intensive Ther. 2014;46(5):361–380. doi: 10.5603/AIT.2014.0060 25432556

[14] O’Connor ME, Prowle JR. Fluid overload. Crit Care Clin. 2015;31(4):803–821. doi: 10.1016/j.ccc.2015.06.013 26410146

[15] Polyzogopoulou E, Velliou M, Verras C, Ventoulis I, Parissis J, Osterwalder J, Hoffmann B. Point-of-Care Ultrasound: A Multimodal Tool for the Management of Sepsis in the Emergency Department. Medicina (Kaunas). 2023;59(6):1180. 37374384 10.3390/medicina59061180 PMC10303071

[16] Tullo G, Candelli M, Gasparrini I, Micci S, Franceschi F. Ultrasound in Sepsis and Septic Shock-From Diagnosis to Treatment. J Clin Med. 2023;12(3):1185. doi: 10.3390/jcm12031185 36769833 PMC9918257

[17] Cecconi M, De Backer D, Antonelli M, Beale R, Bakker J, Hofer C, et al. Consensus on circulatory shock and hemodynamic monitoring. Task force of the European Society of Intensive Care Medicine. Intensive Care Med. 2014;40(12):1795–815. doi: 10.1007/s00134-014-3525-z 25392034 PMC4239778

[18] Wu J, Lu AD, Zhang LP, Zuo YX, Jia YP. Study of clinical outcome and prognosis in pediatric core binding factor-acute myeloid leukemia. Chinese Journal of Hematology. 2019;40(1):52-57 in Chinese. 30704229 10.3760/cma.j.issn.0253-2727.2019.01.010 PMC7351698

[19] Long E, Oakley E, Duke T, Babl FE. Does Respiratory Variation in Inferior Vena Cava Diameter Predict Fluid Responsiveness: A Systematic Review and Meta-Analysis. Shock. 2017;47(5):550–559. doi: 10.1097/shk.0000000000000801 28410544

[20] Kim D-W, Chung S, Kang W-S, Kim J. Diagnostic Accuracy of Ultrasonographic Respiratory Variation in the Inferior Vena Cava, Subclavian Vein, Internal Jugular Vein, and Femoral Vein Diameter to Predict Fluid Responsiveness: A Systematic Review and Meta-Analysis. Diagnostics (Basel). 2021;12(1):49. doi: 10.3390/diagnostics12010049 35054215 PMC8774961

[21] Si X, Xu H, Liu Z, Wu J, Cao D, Chen J, et al. Does Respiratory Variation in Inferior Vena Cava Diameter Predict Fluid Responsiveness in Mechanically Ventilated Patients? A Systematic Review and Meta-analysis. Anesth Analg. 2018;127(5):1157-1164. 29787412 10.1213/ANE.0000000000003459

[22] Alvarado Sánchez JI, Caicedo Ruiz JD, Diaztagle Fernández JJ, Amaya Zuñiga WF, Ospina-Tascón GA, Cruz Martínez LE. Predictors of fluid responsiveness in critically ill patients mechanically ventilated at low tidal volumes: systematic review and meta-analysis. Ann Intensive Care. 2021;11(1):28. doi: 10.1186/s13613-021-00817-5 33555488 PMC7870741

[23] McInnes MDF, Moher D, Thombs BD, McGrath TA, Bossuyt PM, Clifford , and the PRISMA-DTA Group, et al. Preferred Reporting Items for a Systematic Review and Meta-analysis of Diagnostic Test Accuracy Studies: The PRISMA-DTA Statement. JAMA. 2018;319(4):388–396. doi: 10.1001/jama.2017.19163 29362800

[24] Whiting PF, Rutjes AWS, Westwood ME, Mallett S, Deeks JJ, Reitsma JB, et al. QUADAS-2: a revised tool for the quality assessment of diagnostic accuracy studies. Ann Intern Med. 2011;155(8):529–536. doi: 10.7326/0003-4819-155-8-201110180-00009 22007046

[25] Shen LM, Long L, Zhao HT, Ren S. Different indicators predict the accuracy of volume responsiveness in patients with septic shock and myocardial inhibition: comparison of inferior vena cava ultrasound indicators, PiCCO indicators, and CVP. Chinese Journal of Anesthesiology. 2019;39(5):629-632 in Chinese.

[26] Gao S, Zhang Y. Prediction value of bedside inferior vena cava ultrasound index and central venous pressure on volume responsiveness in patients with septic shock. Chinese General Practice. 2021;19(4):581-585 in Chinese.

[27] Yao XQ, Li JG, Ren PP, Wang YL. Evaluation of volume responsiveness of inferior vena cava respiratory variability index measured by bedside ultrasound in patients with septic shock undergoing mechanical ventilation. Imaging Science and Photochemistry. 2020;38(5):811-814 in Chinese.

[28] Zhang HW, Shao M, Shan NB, Li BB. Predictive value of bedside echocardiography combined with Pcv-aCO2 for volume responsiveness in septic shock. Imaging Science and Photochemistry. 2022;40(6):1476-1480 in Chinese.

[29] Chen SJ, Wang YF, Fu XF, Sun MF, Qian MM, Qin C. The relationship between the etiology and transthoracic ultrasound parameters of septic shock and its volume responsiveness and MAP responsiveness after NE reduction. Chinese Journal of Nosocomial Infection. 2023;33(5):688-692 in Chinese.

[30] Zhu WH, Wan LJ, Wan XH, Wang G, Su MX, Liao GJ, et al. Evaluation of brachial artery peak flow variability and inferior vena cava respiratory variability on volume responsiveness. Chinese Critical Care Emergency Medicine. 2016;28(8):713-717 in Chinese.

[31] Lu N, Jiang L, Zhu B, Hav W, Zhao Y, Shi Y, et al. Clinical study of peripheral arterial peak flow variability in evaluating volume responsiveness in patients with septic shock. China Critical Care Emergency Medicine. 2018;30(3):224–229. 29519280 10.3760/cma.j.issn.2095-4352.2018.03.007

[32] Göktürk O, Avci O, Gündoğdu O, İsbi̇r AC, Özdemi̇r Kol, Gürsoy S, et al. Comparison of Pleth Variability Index and Inferior Vena Cava Distensibility as a Perfusion Indicator in Sepsis Patients: An Observational Study. Turkiye Klinikleri Journal of Medical Sciences. 2022;42(2):79-86.

[33] Charbonneau H, Riu B, Faron M, Mari A, Kurrek MM, Ruiz J, et al. Predicting preload responsiveness using simultaneous recordings of inferior and superior vena cavae diameters. Crit Care. 2014;18(5):473. doi: 10.1186/s13054-014-0473-5 25189403 PMC4175634

[34] Lu N, Xi X, Jiang L, Yang D, Yin K. Exploring the best predictors of fluid responsiveness in patients with septic shock. Am J Emerg Med. 2017;35(9):1258–1261. doi: 10.1016/j.ajem.2017.03.052 28363617

[35] Theerawit P, Morasert T, Sutherasan Y. Inferior vena cava diameter variation compared with pulse pressure variation as predictors of fluid responsiveness in patients with sepsis. J Crit Care. 2016;36:246–251. doi: 10.1016/j.jcrc.2016.07.023 27591389

[36] Saber HM, Maraghil SK, Naguib MK, Abd-Elbaset AS, Elkholy MB. Respirophasic Carotid Peak Systolic Velocity Variation as a Predictor of Volume Responsiveness in Mechanically Ventilated Patients with Septic Shock. Egyptian Journal of Critical Care Medicine. 2022,9(2):35-39.

[37] Feissel M, Michard F, Faller J-P, Teboul J-L. The respiratory variation in inferior vena cava diameter as a guide to fluid therapy. Intensive Care Med. 2004;30(9):1834–37. doi: 10.1007/s00134-004-2233-5 15045170

[38] Abdelmaksoud NFS, Elewa GEMA, Sersi MHSA, Nawar DFEA, Eldemerdash AMAY. The use of end-expiratory occlusion test vs. inferior vena cava respiratory variation for the prediction of volume responsiveness in mechanically ventilated patients with sepsis: A comparative study. Egyptian Journal of Anesthesia. 2023;39(1):755–762. doi: 10.1080/11101849.2023.2248738

[39] He H, Pan NF, Zho XY. Application value of bedside ultrasound for assessing volume responsiveness in patients with septic shock. Vojnosanitetski Pregled. 2023, 80(5):439-445

[40] Preau S, Bortolotti P, Colling D, Dewavrin F, Colas V, Voisin B, et ai. Diagnostic Accuracy of the Inferior Vena Cava Collapsibility to Predict Fluid Responsiveness in Spontaneously Breathing Patients With Sepsis and Acute Circulatory Failure. Crit Care Med. 2017,45(3):e290-e297. 10.1097/CCM.0000000000002090 27749318

[41] Zhao J, Wang G. Inferior Vena Cava Collapsibility Index is a Valuable and Non-Invasive Index for Elevated General Heart End-Diastolic Volume Index Estimation in Septic Shock Patients. Med Sci Monit. 2016;22:3843–8. doi: 10.12659/msm.897406 27762259 PMC5085335

[42] Caplan M, Durand A, Bortolotti P, Colling D, Goutay J, Duburcq T, et al. Measurement site of inferior vena cava diameter affects the accuracy with which fluid responsiveness can be predicted in spontaneously breathing patients: a post hoc analysis of two prospective cohorts. Ann Intensive Care. 2020;10(1):168. doi: 10.1186/s13613-020-00786-1 33306164 PMC7732956

[43] Elsaeed AMR, El-Din BME, Taher WAM, Mostafa RH, Saleh AN. Internal jugular vein distensibility variation and inferior vena cava collapsibility variation with fluid resuscitation as an indicator for fluid management in spontaneously breathing septic patients. Ain Shams Journal of Anesthesiology. 2022,14(1):1-7.

[44] Haliloğlu M, Bilgili B, Kararmaz A, Cinel İ. The value of internal jugular vein collapsibility index in sepsis. Ulus Travma Acil Cerrahi Derg. 2017;23(4):294–300. doi: 10.5505/tjtes.2016.04832 28762449

[45] Bortolotti P, Colling D, Colas V, Voisin B, Dewavrin F, Poissy J, et al. Respiratory changes of the inferior vena cava diameter predict fluid responsiveness in spontaneously breathing patients with cardiac arrhythmias. Ann Intensive Care. 2018;8(1):79. doi: 10.1186/s13613-018-0427-1 30073423 PMC6072642

[46] Zhang Z, Xu X, Ye S, Xu L. Ultrasonographic measurement of the respiratory variation in the inferior vena cava diameter is predictive of fluid responsiveness in critically ill patients: systematic review and meta-analysis. Ultrasound Med Biol. 2014;40(5):845–53. doi: 10.1016/j.ultrasmedbio.2013.12.010 24495437

[47] Orso D, Paoli I, Piani T, Cilenti FL, Cristiani L, Guglielmo N. Accuracy of Ultrasonographic Measurements of Inferior Vena Cava to Determine Fluid Responsiveness: A Systematic Review and Meta-Analysis. J Intensive Care Med. 2020;35(4):354–363. doi: 10.1177/0885066617752308 29343170

[48] Huang H, Shen Q, Liu Y, Xu H, Fang Y. Value of variation index of inferior vena cava diameter in predicting fluid responsiveness in patients with circulatory shock receiving mechanical ventilation: a systematic review and meta-analysis. Crit Care. 2018;22(1):204. doi: 10.1186/s13054-018-2063-4 30126449 PMC6102872

[49] Expert Group of the Emergency Medicine Branch of the Chinese Medical Association. Expert consensus on Application of vasopressors in emergency shock. Chinese Journal of Emergency Medicine,2021, 30(8):8.

[50] Hong A, Villano N, Toppen W, Elizabeth Aquije M, Berlin D, Cannesson M, et al. Shock Management Without Formal Fluid Responsiveness Assessment: A Retrospective Analysis of Fluid Responsiveness and Its Outcomes. J Acute Med. 2021;11(4):129–140. doi: 10.6705/j.jacme.202112_11(4).0002 35155089 PMC8743191

[51] Weber MD, Lim JKB, Glau C, Conlon T, James R, Lee JH. A narrative review of diaphragmatic ultrasound in pediatric critical care. Pediatr Pulmonol. 2021;56(8):2471–83. doi: 10.1002/ppul.25518 34081825

[52] Wiedermann CJ. Phases of fluid management and the roles of human albumin solution in perioperative and critically ill patients. Curr Med Res Opin. 2020;36(12):1961–73. doi: 10.1080/03007995.2020.1840970 33090028

[53] Theres H, Binkau J, Laule M, et al. Phase-related changes in right ventricular cardiac output under volume-controlled mechanical ventilation with positive end-expiratory pressure. Crit Care Med. 1999;27(5):953–958. doi: 10.1097/00003246-199905000-00033 10362419

[54] Jiang J J, Yuan H B, Sun P L, Shi XY. Effect of low tidal volume mechanical ventilation combined with positive end-expiratory pressure on intraoperative pulmonary oxygenation in septic patients. Academic Journal of Second Military Medical University. 2007;28(5):570-572.

[55] Oliveira RH, Azevedo LC, Park M, Schettino GP. Influence of ventilatory settings on static and functional haemodynamic parameters during experimental hypovolaemia. Eur J Anaesthesiol. 2009;26(1):66–72. doi: 10.1097/EJA.0b013e328319bf5e 19122555

[56] Liu Y, Wei L, Li G, Yu X, Li G, Li Y. Pulse Pressure Variation Adjusted by Respiratory Changes in Pleural Pressure, Rather Than by Tidal Volume, Reliably Predicts Fluid Responsiveness in Patients With Acute Respiratory Distress Syndrome. Crit Care Med. 2016;44(2):342–351. doi: 10.1097/CCM.0000000000001371 26457754

[57] Corl KA, George NR, Romanoff J, Levinson AT, Chheng DB, Merchant RC, et al. Inferior vena cava collapsibility detects fluid responsiveness among spontaneously breathing critically ill patients. J Crit Care. 2017;41:130–137. doi: 10.1016/j.jcrc.2017.05.008 28525778

[58] Airapetian N, Maizel J, Alyamani O, Mahjoub Y, Lorne E, Levrard M, et al. Does inferior vena cava respiratory variability predict fluid responsiveness in spontaneously breathing patients? Crit Care. 2015;19:400. doi: 10.1186/s13054-015-1100-9 26563768 PMC4643539

[59] Rhodes A, Evans LE, Alhazzani W, Levy MM, Antonelli M, Ferrer R, et al. Surviving Sepsis Campaign: International Guidelines for Management of Sepsis and Septic Shock: 2016. Intensive Care Med. 2017;43(3):304–377. doi: 10.1007/s00134-017-4683-6 28101605

[60] Wu F, Chen H, Chen Q, Zheng R, Liu W. Effect of the completion time of initial 30 mL/kg fluid resuscitation on the prognosis of patients with septic shock. Chinese Critical Care Medicine. 2021;33(7):803-808. 34412748 10.3760/cma.j.cn121430-20201228-00777

[61] Huang R, Dong Y, Zhou Y, Zhang L, Xiong J, Fu J. Time-related association between fluid balance and prognosis in sepsis patients: a cohort study based on MIMIC-IV database. Chinese Critical Care Medicine. 2023;35(11):1182–1187. doi: 10.3760/cma.j.cn121430-20230807-00591 37987129

[62] McIntyre LA, Marshall JC. Intravenous Fluids in Septic Shock - More or Less? N Engl J Med. 2022;386(26):2518-2519. 35709013 10.1056/NEJMe2206160

