# Supplementary material for: Predictive accuracy of changes in the inferior vena cava diameter for predicting fluid responsiveness in patients with sepsis: A systematic review and meta-analysis
Source: PLoS One. 2025 May 9;20(5):e0310462. doi: 10.1371/journal.pone.0310462 (PMC12064207; doi:10.1371/journal.pone.0310462)
Supplement: Attachment — Submitted filename: Response to Reviewers.docx [file pone.0310462.s005.docx]

**Dear Reviewers,**

Happy Thanksgiving Day! Thank you for your thorough review and constructive suggestions regarding our manuscript. We have revised the manuscript in accordance with your comments and have marked all amendments in the revised document. In addition to our point-by-point responses to the reviewers’ comments, we have also made every effort to meet the required editorial standards, ensuring that all changes are easily identifiable. We hope that our revised manuscript fulfills your requirements. If any further action is needed, please do not hesitate to let us know. We look forward to your feedback.

**1. Please improve english grammar and style of the draft**

**Response:** Considering the academic editor’s suggestion, we have engaged a native English speaker to thoroughly revise our language, ensuring the fluency, accuracy, and consistency of our writing. We believe these changes will greatly enhance the quality of our manuscript.

**2. Data Availability Statement**

**Response:** Considering the Reviewer’s suggestion, we have corrected it, and it has been changed to “All data are in the manuscript and/or supporting information files”. Please see the Data Availability Statement in the attachment of *Supplementary Material*.

**3. Literature search section**

**Response:** Considering the Reviewer’s suggestion, we uploaded the files in Excel format of Supplementary information.

**4. Suggest to Give more elaboration on the different observation of outcome effect between dIVC and cIVC from physiological point.**

**Response:** Considering the Reviewer’s suggestion, we modified the Introduction and Discussion accordingly and added the following references. As detailed in the Introduction, page 6, lines 100 -103, see Discussion section, page 14, line 278-287.

**5.** **The reviewer raises an important point** **that vasopressors were not manipulated during fluid challenge in included studies as this might affect the standard reference of CI or CO?**

**Response:** Considering the Reviewer’s suggestion, all members of the research team read all the literatures. We reviewed the full text and found that four literatures did not explicitly mention the use of vasopressors, two literatures excluded patients who were using vasopressors, and the remaining literatures all acknowledged the use of vasopressors during the study period. Subgroup analysis was not feasible due to the limited number of relevant studies. However, this issue is addressed in the Discussion and Limitations sections. Moreover, the details of the literature have been incorporated into Table1 Main characteristics of the eligible studies by the author.

**6. Providing more context on current guidelines for fluid management in sepsis and their limitations.**

**Response:** Considering the Reviewer’s suggestion, we modified the Introduction accordingly. As detailed in the Introduction, page 4, lines 57 -64.

**7. Clarifying the specific advantages of IVC measurements over other methods of assessing fluid responsiveness.**

**Response:** Considering the Reviewer’s suggestion, we modified the Introduction accordingly. As detailed in the Introduction, page 5, lines 74-78, 82-85.

**8. Providing more detail on the search strategy, including full search terms for each database.**

**Response:** Thank you for the comment. We provided a complete search strategy for the PubMed database which showed in Fig 1. We now have elaborated the search strategy in Word format giving the details of Electronic search strategy.

**9. Clarifying the criteria used for assessing study quality and how this information was incorporated into the analysis.**

**Response:** Considering the Reviewer’s suggestion, we used the QUADAS 2 tool as a guide to direct our judgments as to the quality of the included studies. ZH scored and recorded for all references using the QUADAS-2 tool, and then DM reviewed the results. More detailed descriptions in the supplementary Excel file were also provided.

**10. Explaining how discrepancies in data extraction or quality assessment between reviewers were resolved.**

**Response:** Considering the Reviewer’s suggestion, we modified the Materials and Methods accordingly. In case of disagreement between two researchers in the literature screening or data extraction process, the decision was submitted to the third researcher (GYL). As detailed in the Study selection, page 8, lines 142-144.

**11. The rationale for the chosen subgroup analyses could be more explicitly stated.**

**Response:** Considering the Reviewer’s suggestion, we modified the Subgroup analysis accordingly. As detailed in the Subgroup analysis, page 11, lines 210-213.

**12. More detail on the results of the quality assessment for individual studies would be helpful.**

**Response:** Considering the Reviewer’s suggestion, we used the QUADAS 2 tool as a guide to direct our judgments as to the quality of the included studies. More detailed descriptions in the supplementary Excel file were also provided.

**13. A more in-depth exploration of the sources of heterogeneity would strengthen the analysis.**

**Response:** Considering the Reviewer’s suggestion, we modified the Discussion accordingly. see Discussion section, page 12,14-16.

**14. More detailed discussion of the clinical implications of the different thresholds used across studies.**

**Response:** Considering the Reviewer’s suggestion, we modified the Discussion accordingly. see Discussion section, page 16, lines 333-340.

**15. Clearer guidance on how clinicians should integrate these IVC measurements with other clinical parameters when assessing fluid responsiveness.**

**Response:** Considering the Reviewer’s suggestion, we modified the Discussion accordingly. see Discussion section, page 14, lines 279-288.

**16. A more thorough comparison of these findings with other methods of assessing fluid responsiveness in sepsis.**

**Response:** Considering the Reviewer’s suggestion, we modified the Discussion accordingly. see Discussion section, page 12,14-16.
