# Supplementary material for: Predictive accuracy of changes in the inferior vena cava diameter for predicting fluid responsiveness in patients with sepsis: A systematic review and meta-analysis
Source: PLoS One. 2025 May 9;20(5):e0310462. doi: 10.1371/journal.pone.0310462 (PMC12064207; doi:10.1371/journal.pone.0310462)
Supplement: Attachment — Submitted filename: Response to Reviewers-3.docx [file pone.0310462.s006.docx]

**Dear Reviewers,**

Thank you for your thorough review and constructive suggestions regarding our manuscript. We have revised the manuscript in accordance with your comments and have marked all amendments in the revised document. We hope that our revised manuscript fulfills your requirements. If any further action is needed, please do not hesitate to let us know. We look forward to your feedback.

**1. 1. A numbered table of all 885 studies identified in the literature search, including those that were excluded from the analyses.**

**Response:** Considering the Reviewer’s suggestion, we have corrected it, and it has been changed to “supporting information files”. Please see the Data Availability Statement in the attachment of Supporting information-1 of Excle.
