# Supplementary material for: Predictive accuracy of changes in the inferior vena cava diameter for predicting fluid responsiveness in patients with sepsis: A systematic review and meta-analysis
Source: PLoS One. 2025 May 9;20(5):e0310462. doi: 10.1371/journal.pone.0310462 (PMC12064207; doi:10.1371/journal.pone.0310462)
Supplement: Attachment — Submitted filename: Response_to_Reviewers_auresp_3.docx [file pone.0310462.s007.docx]

**Dear Editors,**

Thank you for your thorough review and constructive suggestions regarding our manuscript. We sincerely appreciate the time and effort you dedicated to reviewing and providing feedback. In particular, we highly value the constructive suggestions you made, which were carefully discussed within our team. We fully agree with your recommendations and have made detailed revisions accordingly. These improvements will help enhance the manuscript’s quality and ensure it meets the standards for publication. Once again, thank you for your valuable input, which has greatly contributed to this research. We look forward to your feedback. Below are our point-by-point responses to your comments:

**Methodology:**

**1. The selection criteria differentiating mechanically ventilated from spontaneously breathing patients needs clearer exposition.**

**Response academic editor:** Thank you once again for your constructive feedback. We greatly appreciate and value your insights. In response, we have revised and enhanced the Methodology section. Your valuable input has improved the completeness of the description.

The manuscript has been amended to state: “The inclusion criteria included studies published in Chinese and English that focused on patients with sepsis or septic shock. The research subjects in the original literature should identify whether they are mechanically ventilated or spontaneously breathing patients. Mechanically ventilated patients should collect ventilator parameters.”

**2. The heterogeneity in ventilator parameters across studies requires more detailed analysis**

**Response academic editor:** Thank you for your valuable suggestion. In response, we have revised the Methodology section accordingly. And it has been changed to “For the subgroup analysis, we first compared mechanically ventilated and nonmechanically ventilated individuals. We analyzed various ventilator parameters including TV, PEEP, and threshold, which are mechanically ventilated.”

**3. The rationale for subgroup analysis thresholds should be better justified**

**Response academic editor:** Thank you for your helpful suggestion. In response, we have revised the Methodology section accordingly. The manuscript has been amended to state: “Currently, there is no evidence that a single evaluation parameter or index can be utilized as an endpoint on its own to direct fluid resuscitation in sepsis patients. Our subgroup analysis is based on previous systematic review results, basic ventilator parameters (TV=6-8mL/kg, PEEP=5 cmH2O), and dose selection for fluid resuscitation. Each enrolled patient with septic shock was categorized into the low-volume (200ml), medium-volume (7 mL/kg fluid), or high-volume (above 500 mL) fluid group according to the infusion volume. We conducted subgroup analyses based on TV, PEEP, infusion volume, and diagnostic threshold.”

**Results Presentation:**

**1. Forest plots and funnel plots should be integrated into the main text rather than supplementary materials for better readability**

**Response academic editor:** Thank you for your insightful comment and helpful suggestions. In response, we have revised the forest plots and funnel plots accordingly.

**2.** **The discussion of varying thresholds across studies needs expansion?**

**Response academic editor:** Thank you for your insightful comment and helpful suggestions. In response, we have revised the Results and Discussion section accordingly.

**3.** **The implications of different fluid challenge protocols require more detailed analysis.**

**Response academic editor:** Thank you for your insightful suggestion. In response, we have revised the Results and Discussion section accordingly.

**4. The meta-regression results need clearer presentation, particularly regarding the clinical significance of findings.**

**Response academic editor:** Thank you for your helpful suggestion. In response, we have revised the Results and Discussion section accordingly.

**Discussion Presentation:**

**1.** **The limitations section should be expanded to address:** **Heterogeneity in study protocols, Variations in measurement techniques, Impact of different patient populations.**

**Response academic editor:** Thank you for your helpful suggestion. In response, we have revised the Limitations section accordingly.

**“**First, heterogeneity in measurement protocols primarily arises from differences between mechanically ventilated and non-ventilated patient cohorts. Second, critically ill patients demonstrate significant heterogeneity that those with heart failure, kidney disease, or other comorbidities may exhibit marked variations in fluid resuscitation efficacy. This necessitates the integration of dynamic parameters, such as ultrasound-guided assessments combined with pulse pressure variation (PPV) or fluid challenge tests, when evaluating fluid resuscitation in septic patients. Additionally, blood lactate levels should be combined to comprehensively assess resuscitation outcomes.**”**

**2. Clinical implementation challenges need more thorough discussion.**

**Response academic editor:** Thank you for your thoughtful suggestion. In response, we have revised the Discussion section accordingly.

**3. Specific recommendations for standardizing measurement techniques should be included.**

**Response academic editor:** Thank you for your suggestion. In response, we have revised the Discussion section accordingly.

**4. The implications of different ventilator settings need more detailed analysis.**

**Response academic editor:** Thank you for your suggestion. In response, we have revised the Discussion section accordingly.
